# Tumor-specific intracellular delivery: peptide-guided transport of a catalytic toxin

**DOI:** 10.1038/s42003-022-04385-7

**Published:** 2023-01-17

**Authors:** Curtis A. Allred, Claire Gormley, Indu Venugopal, Shunzi Li, Michael J. McGuire, Kathlynn C. Brown

**Affiliations:** SRI International, Biosciences Division, 140 Research Drive, Harrisonburg, VA 22802 USA

**Keywords:** Peptides, Drug delivery, Targeted therapies

## Abstract

There continues to be a need for cancer-specific ligands that can deliver a wide variety of therapeutic cargos. Ligands demonstrating both tumor-specificity and the ability to mediate efficient cellular uptake of a therapeutic are critical to expand targeted therapies. We previously reported the selection of a peptide from a peptide library using a non-small cell lung cancer (NSCLC) cell line as the target. Here we optimize our lead peptide by a series of chemical modifications including truncations, N-terminal capping, and changes in valency. The resultant 10 amino acid peptide has an affinity of <40 nM on four different NSCLC cell lines as a monomer and is stable in human serum for >48 h. The peptide rapidly internalizes upon cell binding and traffics to the lysosome. The peptide homes to a tumor in an animal model and is retained up to 72 h. Importantly, we demonstrate that the peptide can deliver the cytotoxic protein saporin specifically to cancer cells in vitro and in vivo, resulting in an effective anticancer agent.

## Abstract (web summary)

Peptide modification is used to optimize the transport of saporin specifically to cancer cells in vitro and in vivo, where it exerts anti-cancer actions.

## Introduction

Despite a decline in new cases over the past 30 years, lung cancer is still responsible for ~20% of cancer-related deaths in America^1^. Screening smokers with low-dose spiral computed tomography has improved detection but only 17% of lung cancers are detected at a localized stage. Newer therapies have shifted focus to molecularly guided treatments that are dependent on the genotype and/or phenotype of the tumor^2^. One such therapeutic class is antibody-drug conjugate (ADCs). Antibodies serve as delivery systems by targeting cell-surface receptors whose expression is upregulated in a tumor but has negligible expression on normal cells. Monoclonal antibodies must exhibit high cell specificity to avoid delivery to normal cells. Additionally, they must internalize into the cell to deliver the toxic payload. Because of the antibody’s specificity for cancer cells, drugs too toxic to be administered systemically can be utilized as ADCs. Approval of Kadcyla®^3^, an anti-HER2 antibody conjugated to emtansine, and Adcetris^4,5^, an anti-CD30 antibody conjugated to monomethyl auristatin, reinvigorated ADC development. Since 2017, eight ADCs have received FDA approval^6–8^. However, to date, there are no ADCs approved for the treatment of lung cancer^9^.

Peptides offer an alternative class of cancer-targeting molecules. Peptides rival antibodies in affinity and specificity. They are easier to produce and can be modified regiospecifically to carry a variety of cargos, including macromolecular biotherapeutics. Phage display biopanning has been employed to select peptidic ligands for novel biomarkers present in cancer^10^. We previously isolated a peptide from a phage-displayed peptide library by biopanning on the NSCLC cell line HCC15^11^. This peptide, now referred to as MGS4 (previously HCC15.2) internalizes into ~54% (21/39) of NSCLC lines tested and binds 24% (14/59) of fixed human NSCLC biopsy samples in a tissue microarray. Lack of internalization into immortalized but non-transformed human bronchial epithelial cells, as well as lack of binding to normal adjacent lung tissue samples in the tissue microarrays establishes the specificity of this peptide for cancer cells *vs*. normal lung tissues. As such, MGS4 is a promising targeting molecule for the delivery of cytotoxics to a subset of cancers. Here we optimize our lead peptide by a series of chemical modifications to create a high-affinity peptide that is serum stable and able to deliver cargo intracellularly into cancer cells. The resultant peptide homes to a NSCLC tumor in an animal model. Importantly, we demonstrate that the optimized MGS4 can deliver the cytotoxic protein saporin specifically to cancer cells in vitro and in vivo, resulting in an effective anticancer agent.

## Results

### Monomeric MGS4 binds target cells

**Fig. 1:**
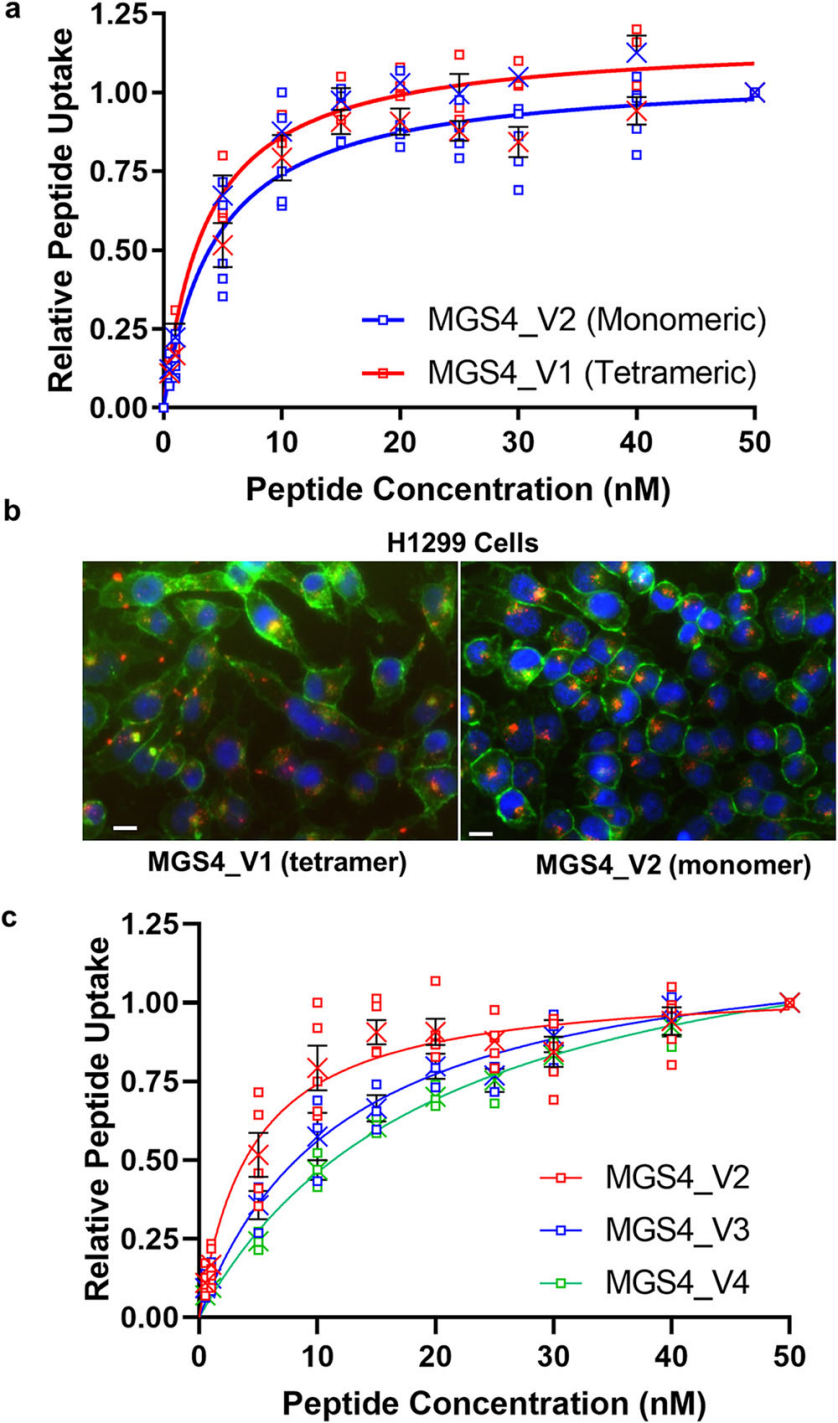
Monomeric and tetrameric MGS4 have similar binding, internalization and subcellular location. **a** Binding and internalization of tetrameric MGS4_V1 and monomeric MGS4_V2 on live H1299 cells in culture. Cells were incubated with the peptide conjugated to streptavidin-phycoerythrin for 1 h at 37 °C. Non-internalized peptide was removed and the cells were analyzed by flow cytometry. **b** H1299 cells were incubated with MGS4_V1 or MGS4_V2 conjugated to streptavidin-Alexa Fluor 555 (red) for 1 h, washed, fixed and counterstained with WGA-Alexa Fluor 488 (green, cell membrane) and DAPI (blue, nuclei) and analyzed by fluorescence microscopy. The scale bar represents 20 µm. MGS4_V1 and MGS4_V2 internalize to a similar degree and localize to a similar destination. **c** Truncated monomeric MGS4 peptides have similar EC50 as the parental full-length peptide. Individual measurements are shown. The mean is shown as an “X” and black error bars represent standard error for a minimum of three experimental replicates (SEM). All original binding data and nonlinear regression analysis of the data are included in the supplementary materials.

MGS4 was initially selected by phage display biopanning of a peptide library on live cells. In the library construction, peptides are genetically fused to the PIII coat protein allowing a single peptide to be displayed in 3-5 copies per phage. As such, multivalent binding is often required. To ascertain whether multivalent binding is required for MGS4 internalization, monomeric (MGS4_V2) and tetrameric (MGS4_V1) peptides were synthesized and labeled as described (Supplementary Figure 1). After incubation of varying peptide concentrations on live cells, surface-bound peptide was removed by low pH washes, as well as trypsinization. Relative internalization was measured by flow cytometry to determine an EC50 value which represents the concentration of peptide producing half-maximal internalization. This measurement is dependent on the affinity of the peptide for its cellular target and the rate of internalization. This is a more accurate representation of the biological situation and useful in evaluating the peptides as drug delivery agents. As expected for receptor-mediated endocytosis, uptake of MGS4 is saturable with increasing concentration. Tetramerization did not significantly alter the EC50 suggesting no apparent change in the affinity of MGS4 with multimerization (Fig. 1a).

Endocytosis is often triggered by receptor multimerization on the cell surface. However, data suggest that MGS4 internalizes in the monomeric format. To verify internalization, the peptide was conjugated to Streptavidin-Alexa Fluor 555 and incubated with live cells. Internalization was determined by confocal fluorescence microscopy. MGS4_V2 showed clear internalization like that observed for MGS4_V1. Both valencies localize into discrete puncta in the perinuclear region (Fig. 1b). Thus, MGS4_V2 binds cancer cells with low nanomolar affinity and delivers cargoes into live cells. As synthesis of the monomeric peptide requires less than half the time and one-quarter of the materials to produce, monomeric MGS4_V2 was used for further optimizations.

### Truncation of MGS4 reveals the minimal binding sequence

To address which amino acids are crucial to cell binding, monomeric MGS4 was synthesized with sequential truncations of amino acids from the termini. The impact of each deletion on internalization was determined (Table 1). Two C-terminal amino acids, alanine and proline, may be truncated with only ~3-5-fold decrease in affinity (MGS4_V3 and MGS4_V4). If the third amino acid, threonine, is removed as well (MGS4_V5), all uptake is lost. Similarly, if the first N-terminal amino acid phenylalanine is truncated (MGS4_V6), uptake is abrogated. While not all amino acids in between are necessarily crucial to binding, MGS4_V4 cannot be truncated further from the termini. While the affinity is decreased by ~3-fold with this truncation, it is advantageous to remove the proline at the C-terminus for practical purposes; proline is susceptible to racemization during peptide coupling, the secondary amine of proline slows coupling of the subsequent amino acid, and proline can reduce overall synthetic yield^12^.

**Table 1:** EC50 for MGS4 peptide variants.

Peptide	Sequence^1^	Valency	EC50 (nM)
MGS4_V1	FHAVPQSFYTAP	Tetramer	3.6 ± 0.49
MGS4_V2	FHAVPQSFYTAP	Monomer	4.4 ± 0.72
MGS4_V3	FHAVPQSFYTA*	Monomer	12.3 ± 1.76
MGS4_V4	FHAVPQSFYT**	Monomer	20.5 ± 1.85
MGS4_V5	FHAVPQSFY***	Monomer	Not Detected^2^
MGS4_V6	*HAVPQSFYT**	Monomer	Not Detected^2^
MGS4_V7	Ac-FHAVPQSFYTAP	Monomer	25.8 ± 6.50
MGS4_V8	Ac-FHAVPQSFYT**	Monomer	20.1 ± 2.55

^1^Asterisks indicated deleted amino acids.

^2^No uptake is observed above background.

All data is presented at Average ± SEM. Primary data and corresponding non-linear fit parameters with statistical analysis are provided in the supplementary material.

### Acetylation protects MGS4 from degradation

Serum stability is often cited as a limitation of peptides with the predominant degradation being cleavage by N-terminal and C-terminal peptidases. The C-terminus is protected from degradation by amidation, a biotinylated amino acid, and a PEG linker (Supplementary Figure 1). The N-terminus, however, contains an unmodified, naturally occurring amino acid phenylalanine, which if removed results in total loss of internalization. Protection from degradation is therefore crucial. Acetylation of the amino terminus protects peptides from N-terminal peptidase while adding minimal steric bulk^13^ However, acetylation reduces the net charge and can alter binding of MGS4 to its cellular receptor.

To address if acetylation is effective in reducing serum degradation of MGS4, acetylated (MGS4_V8) and non-acetylated (MGS4_V4) peptides were dissolved in human serum and incubated for 48 h at 37 °C. The peptides were monitored by analytical HPLC, and products were verified via MALDI TOF/TOF™ MS. Acetylation protects MGS4_V8 from degradation, with only full-length peptide observed (Supplemental Fig. 2). In contrast, none of the starting material of unprotected MGS4_V4 is observed. Instead, a mixture of peptide fragments is detected, none of which correspond to the mass of the starting peptide (Supplemental Table 1 and Supplemental Fig. 2). The major products are shorter fragments corresponding to the loss of the five N-terminal amino acids. Fragments related to QSFYT-PEG11, SFYT-PEG11 and FYT-PEG11 are observed. As we do not observe these cleavage products with MGS4_V8 and we are unable to identify masses that correspond the amino-terminal FHAVP fragment, the degradation products observed with the nonacetylated variant are likely due to aminopeptidase cleavage and not an endoprotease.

To assure acetylation does not affect peptide activity, EC50 of MGS4_V4 and MGS4_V8 were compared. Acetylation has no significant impact on MGS4_V8 internalization compared to non-acetylated MGS4_V4 (Table 1). Similarly, there is a negligible difference between the acetylated full-length peptide MGS4_V7 and the truncated MGS4_V8. Although there is a reduction in the binding of MGS4_V7 compared to MGS4_V2, the affinity as a monomer peptide is still within the useful range for in vivo targeting^14,15^. Thus, acetylation is an effective way to protect the N-terminus of MGS4 without abrogating binding to the cellular target.

### Multimerization increases the EC50 of the MGS4 binding

**Fig. 2:**
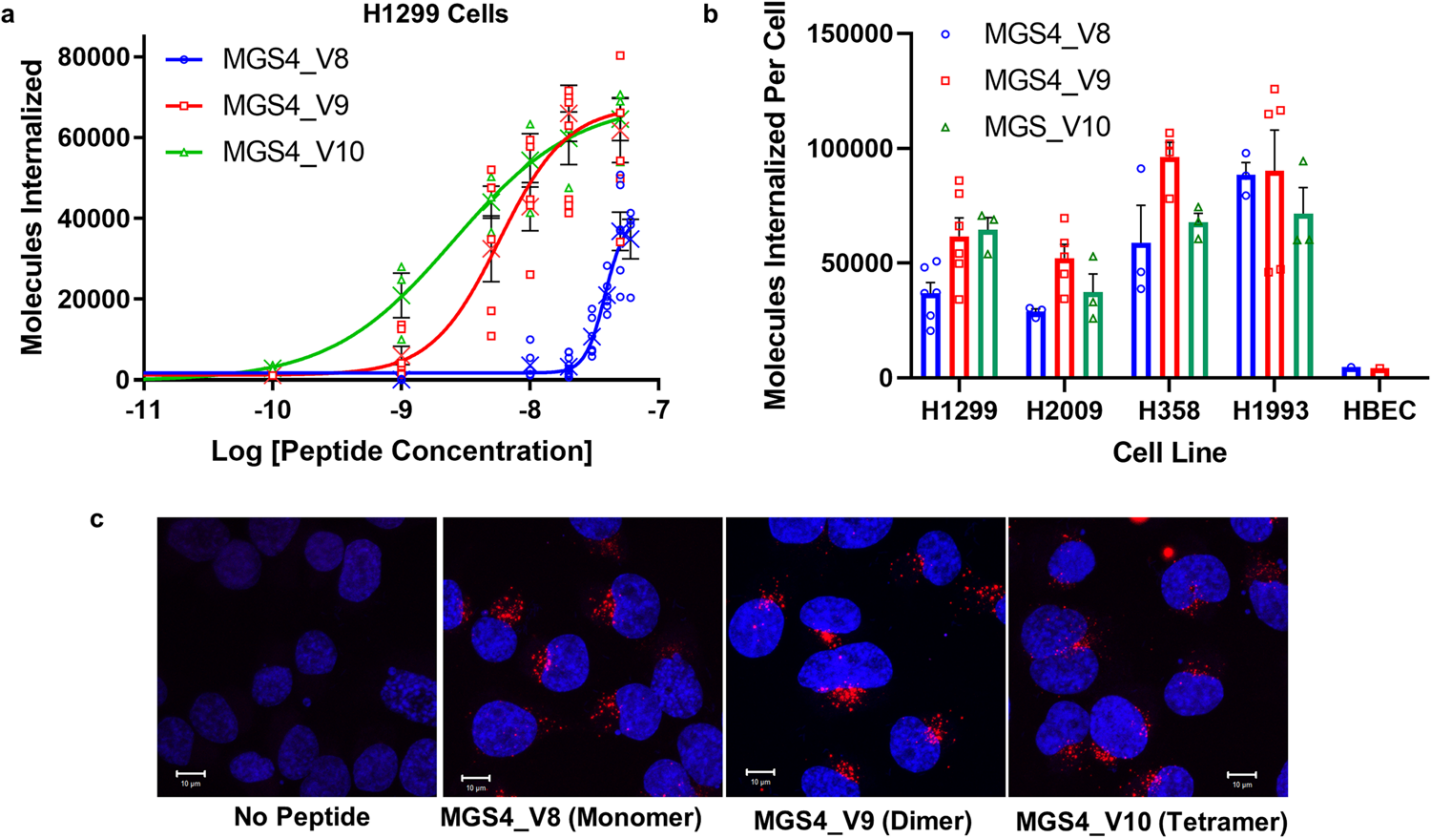
Increased valency of truncated MGS4_V8 decreases EC50 but does not impact absolute update of the peptide or subcellular location. **a** MGS4_V8, MGS4_V9, or MGS4_V10 were conjugated with streptavidin-Alexa Fluor 647 and H1299 cells were incubated with the labeled conjugate for 1 h. Non-internalized peptide was removed, and the mean number of peptides internalized per cell was determined to calculate the EC50. Individual measurements are shown. The mean is shown as an “X”. Error bars, in black, represent standard error measurements and are below the height of the symbols in some cases. **b** The average number of peptide molecules per cell at 50 nM at 1 h was determined on four NSCLC cell lines and one normal human bronchial epithelial cell line (HBEC). Error bars represent SEM and individual data points are shown. **c** H1299 cells were incubated with 50 nM peptide-Streptavidin-Qdot605 for 1 h, removed, and replaced with normal growth media. After 24 h, cells were fixed, and counterstained with DAPI (blue). Representative maximally projected z-stacks for each group reveal no apparent difference in peptide internalization or localization. The scale bar represents 10 µm.

Optimizing MGS4 as a monomer is efficient but the optimal valency of the truncated peptide may be different than that of the full-length peptide. The peptide was synthesized as a monomer (MGS4_V8), dimer (MGS4_V9), and tetramer (MGS4_V10). To obtain quantitative data, we moved from measuring the relative fluorescence of dye-labeled peptide to the determination of the average number of dye molecules internalized per cell. Using this approach, the EC50 for MGS4_V8 on H1299 cells is slightly higher than previously calculated (21 vs 38 nM) but is within the error of the assay. The dimer MGS4_V9 has an EC50 7-fold lower indicating there is a synergistic effect in going from a monomer to a dimer (Fig. 2a and Table 2). However, there is only a 2-fold decrease in going from a dimer to a tetrameric peptide (MGS4_V10). We also calculated the EC50 on three other NSCLC cell lines (Table 2 and Supplementary Figure 3). In all cases the EC50s are the same within experimental error suggesting the affinity of MGS4 is cell type independent. The average number of peptides internalized per cell at saturating conditions varies, with H1993 cells internalizing the highest number of peptides at all three valencies (Table 2). This is likely due to differing levels of receptor expression on the cell type. The average molecules internalized at 50 nM in one hour follows the same trend (Fig. 2b). Additionally, MGS4_V8 and MGS4_V9 retain specificity to NSCLC cell lines; minimal internalization is observed in a normal human bronchial epithelial cell line (Fig. 2b).

**Table 2:** EC50 and peptide uptake of MGS4 in different valencies.

	H1299 Cells		H2009 Cells		H358 Cells		H1993 Cells	
Peptide	EC50 (nM)	Saturation (molecules)	EC50 (nM)	Saturation (molecules)	EC50 (nM)	Saturation (molecules)	EC50 (nM)	Saturation (molecules)
MGS4_V8 (monomer)	38	40,400 ± 5100	38	40,600 ± 2630	34	85,500 ± 39600	37	119,000 ± 57,500
MGS4_V9 (dimer)	5.8	67,900 ± 7980	6.8	53,700 ± 7040	3.9	99,100 ± 11800	4.0	103,000 ± 14,300
MGS4_V10 (tetramer)	2.5	69,200 ± 7650	3.4	41,200 ± 10500	3.5	74,600 ± 8080	1.5	73,600 ± 10,100

While the EC50 decreases with valency, the number of peptides internalized per cell at saturation displays little dependence on valency across the four cell lines. Thus, MGS4 variants of different valencies reach the same maximal uptake albeit at different concentrations (Fig. 2b, Table 2). Similarly, all valencies internalize and traffic to a similar location (Fig. 2c). The increase of the cost in materials and time to synthesize the dimer and tetramer produces an effect of diminishing return. Furthermore, the monomer has the potential to be cloned directly onto proteins for delivery. Taken together, we moved forward with monomeric MGS4_V8.

### MGS4 colocalizes with lysosomal organelle marker

**Fig. 3:**
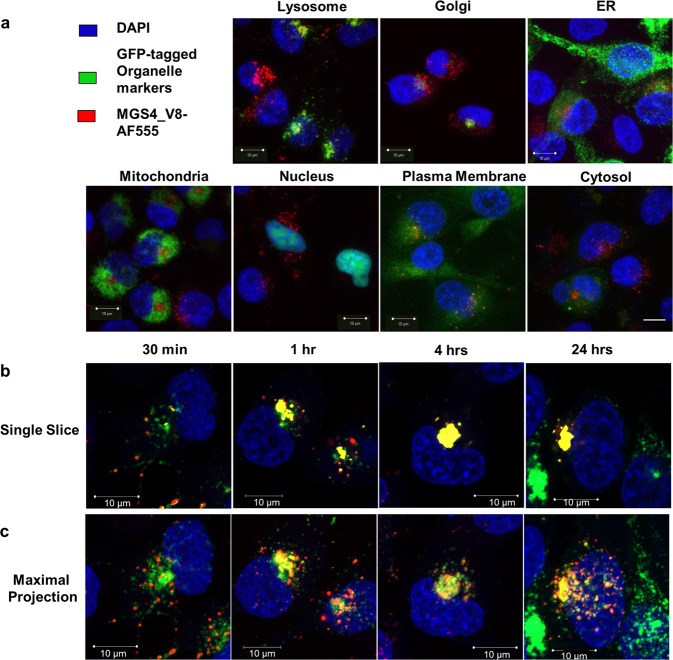
MGS4_V8 traffics to and accumulates in lysosomes over time. **a** H1299 cells were labelled with GFP-tagged organelle-specific proteins for ER, Golgi, lysosome, mitochondria, nucleus, plasma membrane, and cytosol (green). Cells were incubated with 50 nM MGS4_V8-Streptavidin Alexa Fluor 555 (red) for 1 h at 37 °C, then fixed and counterstained with DAPI (blue). MGS4_V8 colocalizes with lysosomes observed as yellow puncta indicated by the red arrows. No significant colocalization is observed with other subcellular organelles. **b** Lysosome labelled H1299 cells (green) were incubated with 50 nM MGS4_V8-Streptavidin Alexa Fluor 555 (red) for 0.5, 1, 4, or 24 h, then washed, fixed, and counterstained with DAPI (Blue). Representative single z-slice images are shown. Peptide-filled vesicles can be seen trafficking to lysosomes at 30 min, with many already colocalizing by 1 h. Most peptide is found within lysosomes by 4 h and retained there at 24 h. **c** Maximally projected, compressed z-stacks from images in panel **b**. The scale bar in all images represents 10 µm.

Internal trafficking of drug conjugates after internalization has important repercussions in efficacy; the cargo must be able to reach its cellular target to produce the desired biological effect. A series of stable organelle-specific, GFP-labelled H1299 cells were generated in which the nuclei, ER, Golgi, lysosome, mitochondria, cytosol, or plasma membrane were labelled. Each cell line was treated with MGS4_V8-Streptavidin Alexa Fluor 555. At one hour, MGS4_V8 colocalization was observed in lysosome-labelled cells, seen as yellow pixels indicated by red arrows in Fig. 3a. MGS4_V8 did not accumulate in the other subcellular locations, nor was it observed on the cell membrane.

### MGS4 accumulates in lysosomes over time

Lysosome-labelled cells (green) were treated with MGS4_V8-Streptavidin Alexa Fluor 555 for 30 min, 1 h, 4 h, or 24 h. Peptide-containing vesicles (red) are seen at 30 min separate from the labeled lysosomes, which by 1 h have started to colocalize with lysosomal vesicles (yellow). MGS4_V8 remains colocalized with lysosomes at 24 h with >70% of the signal colocalized with lysosomes (Fig. 3b). Trafficking, accumulation, and retention in the lysosomes is even more evident in the compressed maximally projected z-stack of the cells (Fig. 3c). Of note, lysosomal regeneration is observed at 24 h as witnessed by increased green signal from the GFP that is not colocalized with previously internalized MGS4_V8.

### MGS4 mediates in vitro intracellular delivery of the protein toxin saporin

**Fig. 4:**
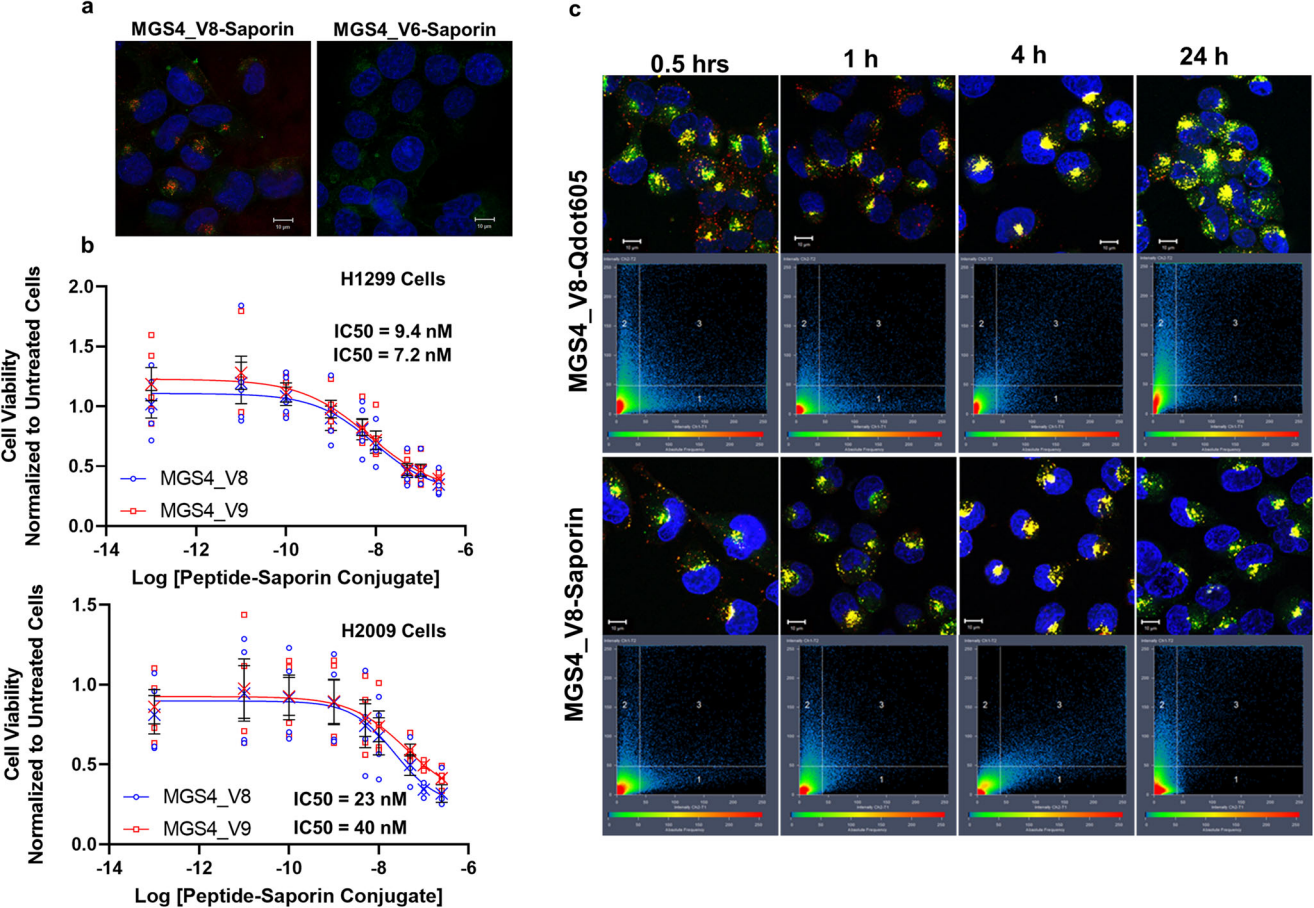
MGS4_V8 effectively delivers active saporin to cancer cells. **a** H1299 cells were incubated biotinylated MGS4_V8 conjugated to streptavidin-saporin for 1 h, then washed, fixed and counterstained with an anti-saporin antibody (red), WGA-AF488 (green) and DAPI (blue). MGS4_V8 successfully delivers saporin into cancer cells while the control peptide, MGS4_V6 cannot. **b** MGS4_V8 and MGS4_V9 saporin-conjugates were serially diluted and incubated with H1299 and H2009 cells for 6 h after which, the MGS4-saporin conjugates were removed and complete growth media returned to the wells. At 72 h, viability was measured. IC50 values are provided in the inset. Individual measurements are shown. The mean is shown as an “X”. Error bars, in black, represent standard error measurements. Non-linear regression analysis is included in the supplementary material. **c** Colocalization time course as before, comparing MGS4_V8-streptavidin-Qdot trafficking to MGS4_V8-saporin trafficking. Pixels are plotted based on intensity in the red channel (x-axis) and the green channel (y-axis). Box 1 represents the population of saporin or Qdots not colocalized with the lysosome. Conversely, box 2 represents lysosomal staining not associated with Qdots or saporin signal. Box 3 contains colocalized pixels, which are falsely colored yellow and represent saporin or Qdots colocalized within the lysosomal compartment. A subpopulation of saporin containing vesicles remain distinct from lysosomes (box 1). The scale bar in all images represents 10 µm.

Saporin is a ribosome-inactivating protein (RIP) which functions by cleaving the ribosomal 28 S rRNA, halting protein synthesis^16–18^. The ribosome-inactivating activity of saporin is catalytic, requiring few molecules to inactivate the ribosomes in a cell. Saporin lacks an internalization domain; it has no tropism for human cells and the toxin is not internalized unless it is linked to a cell-internalizing ligand. To determine if MGS4_V8 delivers an active protein toxin intracellularly, biotinylated peptide was conjugated to streptavidin-labelled saporin. As seen in Fig. 4a, MGS4_V8 mediates saporin internalization into H1299 cells. By contrast, saporin conjugated to the control peptide MGS4_V6 does not enter the cells, demonstrating the requirement of the functional targeting peptide to facilitate its intracellular delivery.

The ability of MGS4_V8-saporin conjugate to induce cell death was determined. MGS4_V8-saporin kills H1299 cells with an IC50 of 9.4 nM. H2009 cells are slightly more resistant, with an IC50 of 23 nM (Fig. 4b). Dimeric MGS4_V9-saporin has an IC50 of 7.2 nM and 40 nM on H1299 and H2009 cells, respectively. Neither MGS4_V8 alone nor MGS4_V9 conjugated to streptavidin without saporin showed any toxicity even up to concentrations of 200 nM (supplemental Fig. 4a). Further, treatment with the inactive MGS4_V6 complexed to saporin does not reach 50% cell death in H1299 and H2009 cells at 200 nM. Normal control HBEC cells do not reach 50% cell viability with either MGS4_V8 or MGS4_V6 (Supplemental Fig. 4b). Although MGS4_V9 has a lower EC50 for both cell types, dimerization does not improve the IC50 or potency of the saporin conjugate, validating the choice of the monomeric peptide as the delivery agent.

### Internalized saporin escapes lysosomal trafficking

The saporin staining (Fig. 4) is strikingly like previous MGS4 staining (Fig. 3c): punctate and perinuclear. However, to induce cell death, the internalized saporin must gain access to the ribosomes in the cytoplasm. The cell viability results suggest that at least some saporin reaches the cytoplasm. This is likely due to endosomal escape mediated by saporin before trafficking to lysosomes. To observe endosomal escape, a time-course was performed to assess saporin colocalization with lysosomes. Biotinylated MGS4_V8 was conjugated to a streptavidin-Qdot605 or to streptavidin-saporin, and the conjugate was incubated with H1299 cells for 30 min, 1 h, 1 h with a 3-hour chase, or 1 h with a 23-hour chase. Both saporin and Qdots traffic to and accumulate in lysosomes (yellow) over time (Fig. 4c). However, there is a discrete population of saporin loaded vesicles (red) that evade trafficking to the lysosome, which are not observed in the Qdot sample. This is especially evident at 1-hour. These non-colocalizing, saporin containing vesicles are evident by the Mander’s coefficient; saporin shows less colocalization compared to Qdots at 1 h (0.334 vs 0.549 respectively) and 24 h (0.657 vs 0.758 respectively) (Table 3). These data suggest that a fraction of saporin escapes from lysosomal trafficking into the cytosol to exert cell killing. As the activity of saporin is catalytic, a fraction is enough for efficacious killing.

**Table 3:** A Subpopulation of saporin-containing vesicles do not colocalize with the lysosome.

Hours	Qdots	Saporin
0.5	0.23 ± 0.024	0.21 ± 0.015
1	0.55 ± 0.015	0.33 ± 0.017
4	0.66 ± 0.026	0.65 ± 0.021
24	0.76 ± 0.018	0.66 ± 0.016

The average and standard error of the weighted Mander’s coefficient calculated from all slices in three images per treatment group. The value represents the population of Qdots or saporin colocalized in the lysosome.

### MGS4_V8 homes to tumors in an in vivo mouse model

**Fig. 5:**
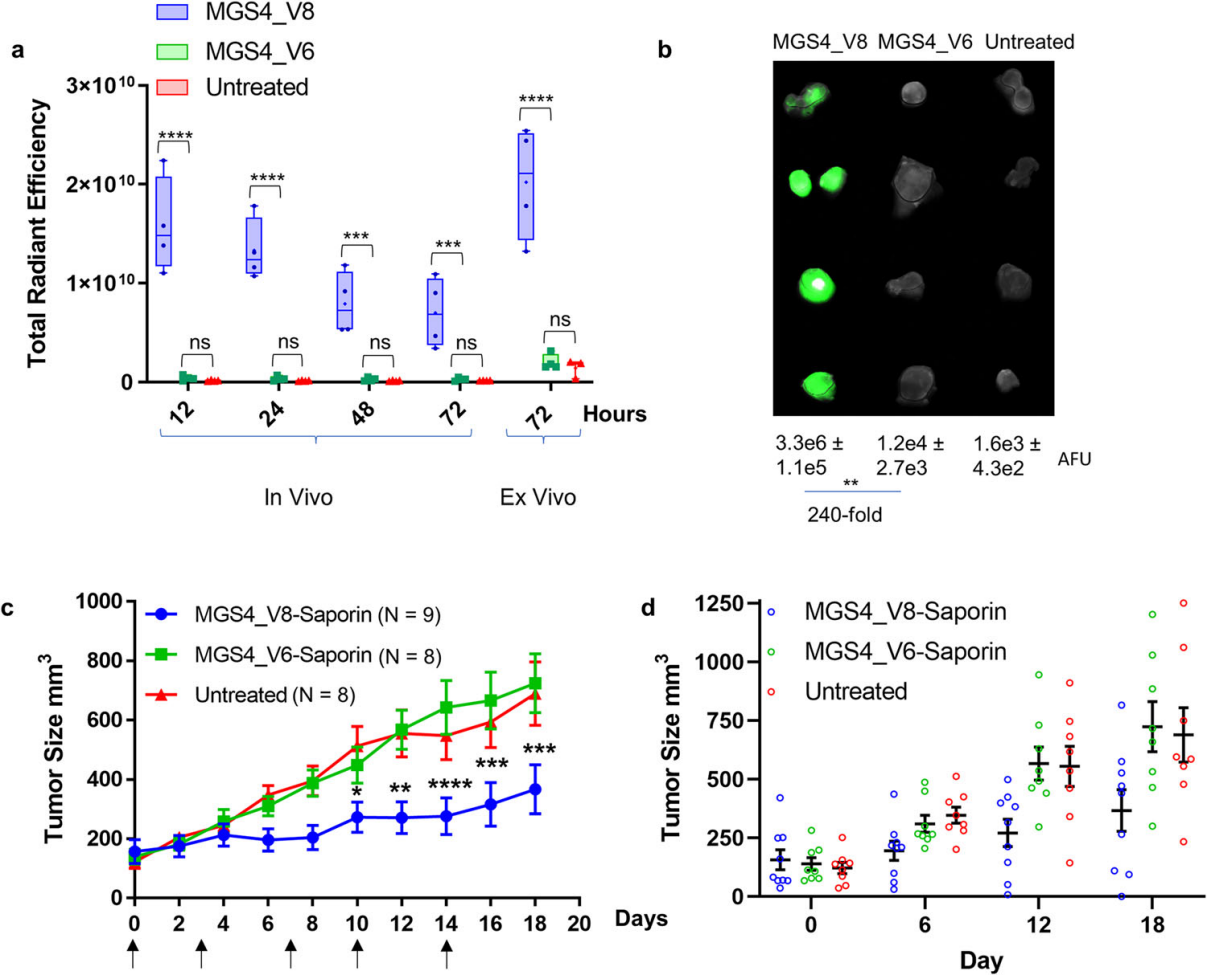
MGS4_V8 homes to xenograft tumor after systemic delivery and can deliver saporin resulting in reduced tumor growth. ** a **H2009 tumor bearing nude mice (*N* = 4) were injected I.V. with MGS4_V8 or MGS4_V6 conjugated to NIR dye Alexa Fluor 750. At 12, 24, 48, and 72 h postinjection, mice were anesthetized and imaged on an IVIS® (Perkin Elmer) to measure total radiant efficiency in each tumor. MGS4_V8 accumulates in tumor 25–39 fold better than control peptide, MGS4_V6. MGS4_V6 accumulation is statistically no different than untreated tumors. Ex vivo NIR imaging of the tumors at the end of the experiment mirrors the data observed in the living animals. Whiskers represent min-to-max values, 25^th^ to 75^th^ percentile are represented by the box, the line shows the median, the + symbol represents the mean, and individual data is shown as a dot. Data for individual animals is included in Supplementary Table 8. **b** Excised tumors from the previous experiment were fixed in PBS + 4% formaldehyde and then imaged again together on a LI-COR^®^ Odyssey. Arbitrary fluorescence units (AFU) were determined for each tumor and the average and SEM are shown below the image. The mean fluorescent intensity is 240-fold greater for MGS4_V8-Alexa Fluor 750 conjugate than the MGS4_V6 control conjugate. **c** Saporin was conjugated to either targeting MGS4_V8 or nontargeting MGS4_V6 and 7.5 µg of the conjugate was injected I.V. into mice bearing subcutaneous tumors. Animals were dosed 2 times per week for 2.5 weeks (indicated by arrows). Tumors were measured every other day. MGS4_V8-saporin clearly slows tumor growth, while nontargeted saporin has no effect compared to untreated animals. Error bars represent SEM, *p-value < 0.05, **p-value < 0.01, ***p-value < 0.001, ****p-value < 0.0001 (two-way ANOVA). **d** Tumor size (mm3) are shown for individual animals at days 0, 6, 12, and 18. Mean value is represented by the horizontal line and the error bars represent standard error. There is no statistical difference between MGS4_V6-Saporin and untreated at any day. At days 12 and 18 MGS4_V8-Saporin is statistically different than untreated (p-values 0.0099 and 0.0029, respectively) and MGS4_V6-Saporin (p-values 0.0069 and 0.0009, respectively).

Use of MGS4 as a delivery agent relies on its ability to target a tumor in an animal. MGS4_V8 and MGS4_V6 (control) were conjugated directly to Alexa Fluor 750. Each conjugate was injected intravenously into immunocompromised mice bearing subcutaneous H2009 tumors, and accumulation of the peptide was measured by near infrared imaging (Fig. 5a). MGS4_V8 tumor homing is observed at 12 h and 85% of that signal is maintained at 24 h. MGS4_V8 signal remains at 48 and 72 h, indicating persistent retention of the dye in the tumor. At all times, MGS4_V8 has 25-40-fold increased signal compared to the control peptide. By comparison, there is no statistically significant difference between the signal resulting from MGS4_V6 and the untreated tumors (background). Tumors imaged ex vivo at 72 h displayed a similar pattern. Tumors were fixed in paraformaldehyde followed by whole tumor imaging on a LI-COR^®^ Odyssey (Fig. 5b). A clear visual difference exists between tumors isolated from mice treated with MGS4_V8 compared to those treated with MGS4_V6 control or untreated. Quantification of fluorescence results in a 240-fold higher signal in the MGS4_V8 treated tumors compared to the MGS4_V6 group. Similar results were obtained when subcutaneous H1299 tumors were established in mice (Supplemental Fig. 5). Together, these data indicated that MGS4_V8 has the specificity, affinity, and stability necessary to target a tumor in vivo. The retention of signal at 72 h is suggestive that MGS4_V8 is internalized into cancer cells within the tumor and the NIR dye remains entrapped.

### MGS4-saporin slows in vivo tumor growth

To establish efficacy in an animal model, H2009 tumors were subcutaneously implanted on the flanks of female nude mice. When H2009 tumors reached ~100 mm^3^, the mice were injected with 7.5 µg of MGS4_V8-saporin or 7.5 µg of acetylated MGS4_V6-saporin (control peptide) via tail vein, 2x/week for a total of 5 injections. MGS4_V8-targeted saporin significantly slowed tumor growth compared to control peptide (Fig. 5c, d). Although the tumor is not eliminated over the course of treatment, the tumor volume remained static for the first 10 days of treatment. By comparison, tumors treated with the non-targeted saporin had increased in size by 3-fold. By day 18, tumors treated with MGS4_V8-targeted saporin were one half the size of those in either control group. The control non-targeted saporin treatment, MGS4_V6-saporin, is no different from the untreated tumors, emphasizing the need for MGS4_V8 for delivery of the saporin.

## Discussion

Tumor-targeting ligands are key components in drug delivery systems for cancer. Although monoclonal antibodies are the most advanced of the clinically available targeting agents, peptides are emerging as a viable alternative^19–21^. Compared to antibodies, peptides have a faster development time and lower production costs because they are readily synthesized allowing for rapid, iterative optimization of their stability, affinity, specificity, solubility, and hydrophobicity. Peptides are also smaller, allowing for deeper penetration in the treatment of solid tumors^22^. Most targeting peptides have focused on naturally occurring peptide ligands or related analogs that bind to receptors upregulated in cancer, e.g., bombesin, luteinizing hormone-releasing hormone, and the tripeptide RGD. ^177^Lu-Dotatate, a radiolabeled somatostatin derivative received FDA accelerated approval in early 2018 and is the first approved peptide-drug conjugate^23^. ANG1005, an LDL receptor-related protein 1 targeting peptide conjugated to paclitaxel is in clinical trials for the treatment of brain metastases^24^ and TH1902, a docetaxel conjugate to a sortilin binding peptide recently entered Phase I clinical trials^25^.

Biopanning of phage-displayed peptide libraries allows for rapid selection of peptides that both bind and initiate internalization specifically in cancer cells^11^. The ability to screen for internalization is key for drug delivery applications as most chemotherapeutics have intracellular targets; ligands and/or receptors that are non-internalizing or have slow cellular uptake are unlikely to be good candidates. This approach is robust and has led to the identification of numerous peptide-targeting agents with high cell specificity^10^. The selection process is unbiased and does not require knowledge of the cell surface repertoire. Although the cellular receptor for MGS4 remains unknown, peptide binding can serve as a surrogate biomarker without knowledge of its cellular target. Studies are ongoing to identify the cellular receptor and establish its expression levels in normal tissues.

The initial hits from these selections are lead compounds, selected as fusions to the pIII coat protein on filamentous phage. Here we have identified the minimal binding domain of MGS4, and chemically optimized it to improve affinity and stability. Peptides are often cited as poor targeting agents compared to antibody candidates due to their limited serum stability and weaker affinities. However, we demonstrate that a monomeric peptide has low nanomolar affinity for its target cells, comparable to antibodies while being 1/100^th^ the size. Modification of both termini stabilizes the peptide in serum. Importantly, MGS4_V8 triggers rapid internalization into the cell. This contrasts with many therapeutic antibodies that have been developed against receptors that have slow internalization rates^26,27^. Surprisingly, the monomeric version internalizes to the same extent as the dimeric and tetrameric peptides and traffics to the same subcellular location. Depending on the cell type, 40,000–100,000 molecules of peptide per cell are internalized in 1 h, reaching intracellular concentration of 40–100 nM^28^. Additional kinetic studies are in progress to determine if the intracellular concentration of the peptide increases with time. Of note, having a series of ligands with a range of affinities for a tumor-specific target and varying rates of internalization could be valuable as a way of addressing the affinity barrier^14^. This phenomenon sometimes hampers penetration into a tumor as the ligand is sequestered rapidly by the first tumor cells it encounters.

MGS4_V8 can be modified to carry a variety of cargos into cells without altering its cell binding or specificity properties. This is clearly indicated in this study as MGS4 delivered proteins (streptavidin, saporin), dyes, and quantum dots. An ideal therapeutic for targeted delivery should have several key features. First, the therapeutic should be cell impermeable except when conjugated to a delivery agent, limiting the potential off target effects if prematurely released from its carrier. Second, it should be effective at low doses as receptor-mediated uptake is unlikely to reach intracellular concentrations that are achievable with cell-permeable small molecule therapeutics. Third, it should escape intracellular vesicles to reach its target. Saporin meets all these criteria. It is a type 1 RIP with no native tropism for mammalian cells and is unable to cross the cell membrane without a delivery vehicle. As an rRNA N-glycosidase enzyme, saporin catalytically inactivates the large ribosomal subunit and does not require stoichiometric amounts to inactivate its target. This activity disrupts protein synthesis in quiescent and actively dividing cells. Saporin can escape intracellular vesicles to reach the cytoplasm and other subcellular organelles^29^. Finally, additional mechanisms of action for saporin independent of its N-glycosidase activity have been identified, increasing its potential cytotoxicity^30–33^. Conjugation of saporin to a cell-specific targeting agent that triggers internalization opens its therapeutic potential.

Coupling of biotinylated MGS4_V8 to saporin-streptavidin conjugate results in a cytotoxic agent. The IC50 is lower than the EC50 for peptide binding and internalization due to saporin’s catalytic nature. One potential concern is saporin’s potential entrapment and degradation in the lysosome. Endocytosis of the MGS4_V8 peptide results in lysosomal accumulation, which increases with time. At 30 min, labeled MGS4_V8 is not colocalized with the lysosome but is seen in punctate vesicles that are likely endosomal compartments. By 1 h, 70% of the peptide is localized in the lysosome. Yet, MGS4_V8-saporin conjugate demonstrates cell cytotoxicity, likely due to a portion of saporin escaping lysosomal trafficking. Our microscopy data indicate that there is a discrete subpopulation MGS4_V8-saporin that is not colocalized with the lysosome, consistent with saporin’s ability for endosomal escape. This population is likely small as diffuse cytoplasmic staining is not observed but high enough to affect cell viability. Approaches to further facilitate endosomal/lysosomal escape of saporin may improve the efficacy of MGS4-saporin conjugates^34–36^. Importantly, MGS4_V8 has potential as a peptide-drug conjugate in which lysosomal trafficking of drug conjugates allows for the use of acid labile or cathepsin cleavable linkers to release the drug^21^.

Saporin-conjugates have served as useful biological tools but also have potential as clinical therapeutics^16–18^. Saporin has been conjugated to differing targeting moieties, the majority of which are antibody-based^37^. We demonstrate that MGS4_V8-saporin has anti-tumor efficacy in a mouse model. Use of the non-targeting MGS4_V6 peptide results in no reduction of tumor size compared to untreated control. Although a complete pharmacokinetic and toxicity profile has yet to be performed, no gross toxicity was observed. Full biodistribution studies and toxicology are needed to address off-target effects. Additionally, improvements to the MGS-saporin linker are required. However, these data demonstrate that MGS4_V8 can deliver active saporin to a tumor in an animal when delivered intravenously and support further exploration in preclinical models.

Two Phase I/II clinical trials of immuno-saporin conjugates have been completed. The first employed mouse anti-CD30 antibody-saporin conjugate for the treatment of refractory Hodgkin lymphoma^38^. Despite promising clinical responses, patients developed an immune response against both the antibody and toxin. Vasculature leak syndrome (VLS) was observed as a dose-limiting toxicity. A second trial using a bispecific antibody against CD22 and saporin demonstrated fewer side effects, including a reduction in VLS and no antisaporin immune response^39^. Both trials were conducted with early-generation mouse antibodies. While there have been no recent clinical trials of saporin conjugates for cancer treatment, saporin conjugated to substance P for pain management was found to safe in a Phase I clinical trial (NCT02036281) and was successfully used for pain management in dogs with bone cancer^40^.

An expanding number of targeting agents against cancer biomarkers has expanded interest in immunotoxins^41–47^. Engineering of toxins to remove immunogenic epitopes and VLS activity continues to progress^48–51^. In addition to saporin, the plant-derived RIP toxins gelonin, ricin, and pokeweed antiviral protein have been utilized in immunotoxins^52^, including the completion of a Phase I trial of an anti-CD33-gelonin immunoconjugate for the treatment of refractory myeloid malignancies^53^. Additionally, toxins from bacterial source, such as *Pseudomonas* Exotoxin A and Diphtheria toxin have been utilized as therapeutic moieties for immunotoxins^54–57^. Ontak, an interleukin-2- diphtheria toxin fusion protein was FDA-approved for cutaneous T cell lymphoma but has since been discontinued due to production issues. Tagraxofusp is an IL-3 targeted diphtheria toxin that was approved for clinical use in 2018 for the treatment of blastic plasmacytoid dendritic cell neoplasm^58^. Lumoxiti, a CD22-PE38 conjugate received FDA approval in 2018 for relapse or refractory hairy cell leukemia^59^. Durable complete response in 30% of patients was achieved in the Phase III trial.

In sum, we have developed a high-affinity, cancer-specific peptide with the ability to deliver active protein toxins to NSCLC cells in vitro and in vivo. This peptide can serve as an antibody replacement in traditional ADCs, reducing costs and production time. The ability to chemically conjugate cargo in a chemically defined fashion is an advantage over ADC. As the parental MGS4 peptide binds multiple cancer cell types beyond NSCLC, we anticipate MGS4_V8 will find expanded utility in other cancer types. MGS4 peptide variants can be incorporated into a variety of different protein toxins, either by genetic engineering or chemical conjugation, expanding its value.

## Materials and Methods

### Chemical and reagents

NovaPEG Rink Amide resin and FMOC-Glu(biotinyl-PEG)-OH were purchased from NovaBiochem^®^(Millipore Sigma, Billerica, MA). 2-(6-Chloro-1H-benzotriazole-1-yl)-1,1,3,3-tetramethylaminium hexafluorophosphate (HCTU), N,N-Dimethylmethanamide, N-Methylmorpholine, 2,2,2-Trifluoroacetic acid, and all FMOC amino acids were purchased from Gyros Protein Technologies (Tucson, AZ). FMOC-NH-(PEG)_11_-COOH (C_42_H_65_NO_16_) was purchased from Polypure (Oslo, Norway). Triisopropylsilane and 1,2-Ethanedithiol were purchased from Sigma Aldrich (Livermore, CA). Piperidine was purchased from Alfa Aesar (Tewksbury, MA), and acetonitrile, dichloromethane, diethyl ether through VWR (Radnor, PA).

### GFP-labeled constructs

The following GFP-labeled constructs were utilized: Plasma membrane label Src-myrisylated-GFP, pmyr GFP (Addgene plasmid # 50528) was a gift from Kenneth Yamada. Golgi label beta-1,4-galactosyltransferase 1-GFP, PA-GFP (Addgene plasmid # 57164), lysosome Label Lamp-1-GFP, Emerald-lysosome-20 (Addgene plasmid # 56476). ER label SigPep-eGFP-KDEL, mEGFP-endoplasmic reticulum (Addgene plasmid # 56455), mitochondria label mitochondrial import receptor subunit translocase of outer membrane 20 kDa subunit-GFP, mEmerald-TOMM20-N-10 (Addgene plasmid # 54282), nucleus label SV40 NLS-GFP, mEmerald-nucleus 7 (Addgene plasmid # 54206), and cytoplasm label Argonaut 3 isoform A-GFP, mEmerald-EIF2C3-C18 (Addgene plasmid # 54078) were gifts from Michael Davidson. Golgi label Tyrosyl protein sulfotransferase 2, TPST2-EGFP was a gift from David Stephens (Addgene plasmid # 66618). Selections were performed in G418 or by limiting dilution.

### Peptide synthesis

Peptide synthesis, cleavage, purification, and multimerization was accomplished by solid-phase synthesis as previously published^60^. Multimeric peptides were synthesized on a lysine (dimer) or trilysine (tetramer) core. The peptide structures (Supplementary Figure 1) are provided in supplemental materials. Peptide masses were confirmed by MALDI/TOF mass spectrometry and >95% pure as determined by analytical HPLC.

### Cell binding and internalization assays

Cell lines were provided by John Minna and Adi Gazdar (UT Southwestern Medical Center) or purchased from ATCC^®^ and maintained in RPMI supplemented with glutamine + 5% fetal bovine serum (Gemini Bio-Products, Sacramento, CA). Cells were genotyped (Bio-synthesis, Lewisville, TX) to confirm identify and evaluated for Mycoplasma infection monthly.

Biotinylated peptide was conjugated to streptavidin-R-phycoerythrin or streptavidin-Alexa Fluor 647 (1:1 molar ratio) for 30 min. The open binding sites on streptavidin were quenched with RPMI 1640 and diluted to the indicated concentration. Tumor cells were grown to 90% confluency in a 12 well plate, then incubated with 500 µl peptide-dye conjugate at 37 °C. After 1 h, peptide was removed, and the cells were washed 3x with PBS (137 mM NaCl, 2.7 mM KCl, 10 mM Na_2_HPO_4_, 1.8 mM KH_2_PO_4_, pH 7.4), 2x with 0.1 M HCl-glycine pH2.2 in 0.9% NaCl, and 1x PBS rinse. Cells were removed by trypsinization. Flow cytometry was performed on a BD FACSCelesta, and data were analyzed on Flowing software. Cells were gated based on the forward and side scatter to include only viable cells and a minimum of 10,000 events were counted. A region containing <5% of the cells in the negative control was established, and relative peptide uptake by the mean fluorescence intensity of that population^11^. For absolute peptide uptake per cell, a standard curve was generated using Quantum™ Alexa Fluor 647 microspheres (Bangs Laboratory, Fishers, IN). A representative linear regression showing the correlation of molecule equivalents soluble fluorophores (MESF) vs the mean fluorescence intensity (MFI) is shown in Supplemental Figure 6. Cells were gated based on the forward and side scatter to include only viable cells and a minimum of 10,000 events were counted. MFI was determined at 50% at peak height. Molecules internalized per cell were determined by the standard curve relating MESF to MFI and divided by the number dye molecules/MGS conjugate. An example is shown in supplemental Figure 7. GraphPad Prism® was used for non-linear regression curve fitting to calculate an EC50. Parameters and statistical analysis are provided (Supplementary Tables 2–6). Experiments were repeated a minimum of three times.

### Confocal microscopy

Plasmids with organelle-specific markers labeled with GFP were purchased from Addgene (Cambridge, MA) and electroporated into H1299 cells. GFP-labeled tumor cells were plated on 8-well chamber slides. Biotinylated peptide was conjugated to streptavidin-Alexa Fluor 555 (1:1) for 30 min at RT and quenched with RPMI, then added to the wells at 50 nM. After 1-hour incubation cells were washed as described. Cells were fixed in 2% formaldehyde. EverBrite™ (Biotium, Freemont, CA) mounting media containing DAPI was used. When indicated, the cell surface was counterstained using wheat germ agglutin (WGA) labeled with Alexa Fluor 488. Microscopy was acquired on a Zeiss LSM 700 with a Pln Apo 63x/1.4 oil DIC III objective. Images were processed using Zen software.

The time course for lysosomal accumulation was performed by imaging at indicated time points using biotinylated peptide conjugated to Streptavidin-Alexa Fluor 555, Streptavidin-Qdot605, or streptavidin-saporin. Saporin was detected using anti-saporin rabbit polyclonal antibody AB-41AP (1:100 dilution) (Advanced Targeting Systems Bio, San Diego, CA). Thresholds were established for Ch1, representing the peptide in the red channel (75), and Ch2, representing the lysosome in the green channel (55). Each pixel of a single slice was evaluated for passing the threshold in red (box 1), green (box 2), or both (box3) for colocalization. Mander’s coefficients are calculated for each slice with 0 indicating no colocalization and 1 being complete colocalization.

### In vitro saporin delivery

Biotinylated peptide was conjugated to streptavidin-saporin (Advanced Targeting Systems Bio, San Diego, CA) in a 1:1 molar ratio. Increasing doses of peptide-drug conjugate in triplicate were incubated on the cells for 6 h at 37 °C. The drug was removed and replaced with complete growth media. After 72 h, cell viability was measured using CellTiter-GLO^®^ (Promega, Madison, WI). Cell viability was normalized to untreated cells. IC50s were calculated using GraphPad Prism® using log(agonist) vs. response–Variable slope (four parameters). Data from individual experiments as well as the parameters and statistical analysis are provided in the supplementary material. A minimum of four biological replicates were performed for each variant and cell line.

### In vivo delivery

Animal experiments were approved by SRI International’s Institutional Animal Care and Use committee (Animal Welfare Assurance Number A3025-01, protocol 14008). H2009 cells (10^6^) or H1299 cells (10^6^) were implanted subcutaneously on the flank of female Nu/Nu mice (Jackson Laboratory, Bar Harbor, ME). When tumors reached 100 mm^3^, in vivo experiments were initiated. For imaging, the indicated peptides were conjugated directly to Alexa Fluor 750 near-infrared dye (Supplemental Fig. 1). Four animals per group were used. Peptides were injected into the lateral tail vein for a total dose 15 µgs/mouse delivered in 100 µL. At set period of times, animals were anesthetized and imaged on an IVIS® (Perkin Elmer). Regions of interest were drawn around the tumor and the total radiant efficiency was measured. For therapeutic experiments, we chose to use the H2009 tumor model as it has a higher tumor take rate and more consistent growth rate than the H1299 model. Biotinylated MGS4_V8 (N = 9) or MGS4_V6 (N = 8) were conjugated to streptavidin-saporin and administered via tail-vein injection (7.5 µg/100 µl) 2x/week for 2.5 weeks for a total of 5 treatments. Nontreated animals (N = 8) served as the control. Tumor size was measured with calipers every other day and volume was calculated as (π/6)(l*w)^3/2^. Statistical analysis was performed on GraphPad Prism®.

### Statistics and reproducibility

For all EC50 determinations, each peptide variant was tested on the indicated cell line with a minimum of 3 biological replicates and analyzed individually by flow cytometry. The standard error measurement for each concentration is indicated by error bars in the figures. For experiments in which absolute number of molecules internalized was measured at different concentrations, EC50 were determined by GraphPad Prism® using nonlinear regression curve fitting for log(agonist) vs. response–Variable slope (four parameters). For the truncation experiments, EC50 were determined using one-site-specific binding. Similarly, IC50s were calculated using GraphPad Prism® using log(agonist) vs. response–Variable slope (four parameters). A minimum of four biological replicates were performed for each variant and cell line. Data for individual experiments are included in supplementary files. Parameters and statistical analysis are provided in Supplementary Tables 2–7.

For in vivo therapeutic experiments, animal group sizes of *N* = 9 for MGS4_V8, *N* = 8 for MGS4_V6 and *N* = 8 for untreated were used. Tumors were measured by an independent researcher with no knowledge of the treatment groups. SEM are represented as error bars on the figures. For tumor imaging, 4 animals were used per group, and error bars represent standard error measurements. For all in vivo experiments, data for individual mice are included in Supplementary Table 9. Statistical significance was determined by two-way ANOVA using Tukey’s multiple comparison test. *P* values < 0.05 are considered significant and are represented on the figures as: *p value < 0.05, **p value < 0.01, ***p value < 0.001, ****p value < 0.0001.

### Reporting summary

Further information on research design is available in the Nature Portfolio Reporting Summary linked to this article.

## Supplementary information

Supplementary Information

Description of Additional Supplementary Files

Supplementary Data 1

Reporting Summary
