# Supplementary material for: Tumor-specific intracellular delivery: peptide-guided transport of a catalytic toxin
Source: Commun Biol. 2023 Jan 17;6:60. doi: 10.1038/s42003-022-04385-7 (PMC9845330; doi:10.1038/s42003-022-04385-7)
Supplement: Supplementary file 1 — Supplementary Information [file 42003_2022_4385_MOESM1_ESM.pdf]

Supplementary Materials

Allred et. al.

Tumor-Specific Intracellular Delivery: Peptide-Guided Transport of a Catalytic Toxin

Supplemental Table 1. Acetylation Protects MGS4 from Degradation in Human Serum

MGS4 Variant	Theoretical Mass	Sample	Observed Mass (MH+)	Δ Mass	% of Peak	Corresponding Peptide
Non Acetylated	1794	Starting Material	1795	0	99.9%	FHAVPQSFYT-PEG_{11}
		0 Hours	1795	0	91.8%	FHAVPQSFYT-PEG_{11}
			1821	+26	8.2%	?
		48 Hours	1488	-307	26.6%	?
			1503	-292	9.1%	?
			1248	-547	35.1%	†(Na^+)pyroESFYT-PEG_{11}
			1116	-679	8.8%	SFYT-PEG_{11}
			1028	-767	12.8%	FYT-PEG_{11}
Acetylated	1836	Starting Material	1837	0	93.5%	AcFHAVPQSFYT-PEG_{11}
			1868	+31	6.5%	Starting Contaminant
		0 Hours	1837	0	95.1%	AcFHAVPQSFYT-PEG_{11}
			1868	+31	4.9%	Starting Contaminant
		48 Hours	1837	0	93.4%	AcFHAVPQSFYT-PEG_{11}
			1868	+31	6.6%	Starting Contaminant

† pyroglutamate (pyroE) is spontaneously formed from N-terminal glutamine (Q)

Supplementary Table 2. Non-linear Regression Analysis of EC50 for MGS4 Variants V1-V8 on H1299 Cells using Normalized Binding						
One-Site Specific Binding						
Best-fit values	**MGS4_V1**	**MGS4_V2**	**MGS4_V3**	**MGS4_V4**	**MGS4_V7**	**MGS4_V8**
Bmax	1.171	1.065	1.247	1.402	1.470	1.423
Kd	3.616	4.375	12.26	20.47	25.84	20.13
Std. Error						
Bmax	0.03084	0.03709	0.06150	0.05498	0.1803	0.07781
Kd	0.4908	0.7232	1.756	1.846	6.500	2.550
95% CI (asymptotic)						
Bmax	1.108 to 1.234	0.9902 to 1.139	1.121 to 1.373	1.289 to 1.515	1.109 to 1.832	1.243 to 1.602
Kd	2.611 to 4.621	2.921 to 5.829	8.658 to 15.85	16.69 to 24.26	12.81 to 38.87	14.25 to 26.01
Goodness of Fit						
Degrees of Freedom	28	48	28	28	54	8
R squared	0.9574	0.9027	0.9594	0.9865	0.8484	0.9924
Sum of Squares	0.1503	0.5054	0.1267	0.04189	0.9655	0.008249
Sy.x	0.07327	0.1026	0.06728	0.03868	0.1337	0.03211
Number of points						
# of X values	60	60	60	60	60	60
# Y values analyzed	30	50	30	30	56	10

Supplementary Table 3. Non-linear Regression Analysis of EC50 for MGS4 Variants V8-V10 on H1299 Cells using Absolute Uptake of Peptide			
log(inhibitor) vs. response -- Variable slope (four parameters)			
Best-fit values	**MGS4_V8**	**MGS4_V9**	**MGS4_V10**
Bottom	1740	1206	-243.9
Top	40400	67869	68966
LogIC50	-7.417	-8.239	-8.598
HillSlope	5.657	1.650	0.9116
IC50	3.827e-008	5.773e-009	2.525e-009
Span	38659	66664	69210
Std. Error			
Bottom	1238	4196	3849
Top	5097	7976	7645
LogIC50	0.03011	0.1117	0.1623
HillSlope	1.721	0.7536	0.2766
Span	5538	9882	9522
95% CI (profile likelihood)			
Bottom	-1047 to 4231	-8295 to 9338	-9362 to 7330
Top	33770 to 62360	56651 to 97516	57658 to 106325
LogIC50	-7.466 to -7.303	-8.518 to -7.912	-8.922 to -7.953
HillSlope	2.897 to 10.55	0.7002 to 3.896	0.4438 to 1.654
IC50	3.416e-008 to 4.973e-008	3.033e-009 to 1.224e-008	1.198e-009 to 1.113e-008
Goodness of Fit			
Degrees of Freedom	45	39	17
R squared	0.8567	0.7806	0.9312
Sum of Squares	1477844510	8609062620	974335981
Sy.x	5731	14857	7571
Number of points			
# of X values	140	126	126
# Y values analyzed	49	43	21

Supplementary Table 4. Non-linear Regression Analysis of EC50 for MGS4 Variants V8-V10 on H2009 Cells using Absolute Uptake of Peptide			
log(inhibitor) vs. response -- Variable slope (four parameters)			
Best-fit values	**MGS4_V8**	**MGS4_V9**	**MGS4_V10**
Bottom	-497.7	1793	-368.8
Top	40633	53679	41200
LogIC50	-7.424	-8.168	-8.471
HillSlope	3.175	1.755	0.7483
IC50	3.771e-008	6.787e-009	3.383e-009
Span	41131	51886	41569
Std. Error			
Bottom	917.2	3435	3002
Top	2627	7039	10525
LogIC50	0.02513	0.1125	0.3658
HillSlope	0.3853	0.8431	0.3872
Span	3020	8448	12014
95% CI (profile likelihood)			
Bottom	-2357 to 1281	-7079 to 8555	-23209 to 5096
Top	35771 to 48593	43121 to 126943	29437 to ???
LogIC50	-7.476 to -7.352	-8.429 to -7.182	-9.084 to ???
HillSlope	2.454 to 4.107	0.4951 to 6.683	0.1107 to 2.185
IC50	3.343e-008 to 4.441e-008	3.727e-009 to 6.572e-008	8.233e-010 to ???
Goodness of Fit			
Degrees of Freedom	20	32	20
R squared	0.9815	0.7768	0.8413
Sum of Squares	81913571	4281724366	910840852
Sy.x	2024	11567	6748
Number of points			
# of X values	156	130	130
# Y values analyzed	24	36	24

Supplementary Table 5. Non-linear Regression Analysis of EC50 for MGS4 Variants V8-V10 on H358 Cells using Absolute Uptake of Peptide			
log(inhibitor) vs. response -- Variable slope (four parameters)			
Best-fit values	**MGS4_V8**	**MGS4_V9**	**MGS4_V10**
Bottom	-220.5	-584.5	-1400
Top	85477	99144	74615
LogIC50	-7.470	-8.408	-8.456
HillSlope	1.829	1.137	0.7758
IC50	3.388e-008	3.907e-009	3.502e-009
Span	85697	99728	76015
Std. Error			
Bottom	6352	5758	3144
Top	39620	11770	8076
LogIC50	0.2536	0.1487	0.1428
HillSlope	1.158	0.3717	0.1827
Span	41572	14405	9941
95% CI (profile likelihood)			
Bottom	-14407 to 12246	-14115 to 10331	-10981 to 4370
Top	52274 to ???	82143 to 173042	62742 to 119322
LogIC50	-7.750 to ???	-8.703 to -7.707	-8.719 to -7.760
HillSlope	0.4689 to 5.388	0.4909 to 2.466	0.4029 to 1.219
IC50	1.776e-008 to ???	1.983e-009 to 1.961e-008	1.911e-009 to 1.737e-008
Goodness of Fit			
Degrees of Freedom	28	33	17
R squared	0.6777	0.8620	0.9672
Sum of Squares	8494267711	8189420034	445308201
Sy.x	17417	15753	5118
Number of points			
# of X values	144	120	120
# Y values analyzed	32	37	21

**Supplementary Table 6. Non-linear Regression Analysis of EC50 for MGS4 Variants V8-V10 on H993 Cells using Absolute Uptake of Peptide**			
log(inhibitor) vs. response -- Variable slope (four parameters)			
Best-fit values	**MGS4_V8**	**MGS4_V9**	**MGS4_V10**
Bottom	-810.7	1440	-1180
Top	118643	102586	73570
LogIC50	-7.427	-8.398	-8.823
HillSlope	1.706	1.703	0.9499
IC50	3.740e-008	3.999e-009	1.503e-009
Span	119453	101146	74750
Std. Error			
Bottom	4192	10753	6840
Top	57461	14315	10082
LogIC50	0.2659	0.1795	0.2282
HillSlope	0.7580	1.148	0.4521
Span	58579	19818	13754
95% CI (asymptotic)			
Bottom	-9350 to 7728	-20437 to 23316	-15610 to 13251
Top	1599 to 235686	73463 to 131710	52300 to 94840
LogIC50	-7.969 to -6.886	-8.763 to -8.033	-9.304 to -8.341
HillSlope	0.1619 to 3.250	-0.6320 to 4.038	-0.003990 to 1.904
IC50	1.075e-008 to 1.302e-007	1.725e-009 to 9.271e-009	4.962e-010 to 4.555e-009
Span	132.7 to 238774	60827 to 141465	45732 to 103767
Goodness of Fit			
Degrees of Freedom	32	33	17
R squared	0.8423	0.6483	0.8544
Sum of Squares	5607042968	35209281402	2852300555
Sy.x	13237	32664	12953
Number of points			
# of X values	88	80	80
# Y values analyzed	36	37	21

Supplementary Table 7. Non-linear Regression Analysis of IC50 for MGS4-Saporin Conjugates		
**H1299 Cells**		
**log(inhibitor) vs. response -- Variable slope (four parameters)**		
Best-fit values	**MGS4_V8**	**MGS4_V9**
Bottom	0.2439	0.2782
Top	1.032	1.227
LogIC50	-8.025	-8.144
HillSlope	-0.6206	-0.5722
IC50	9.436e-009	7.171e-009
Span	0.7885	0.9488
95% CI (asymptotic)		
Bottom	-0.2569 to 0.7448	-0.1568 to 0.7132
Top	0.8904 to 1.175	1.090 to 1.364
LogIC50	-9.137 to -6.913	-8.995 to -7.294
HillSlope	-1.431 to 0.1898	-1.125 to -0.01983
IC50	7.286e-010 to 1.222e-007	1.012e-009 to 5.080e-008
Span	0.2134 to 1.364	0.4378 to 1.460
Goodness of Fit		
Degrees of Freedom	50	41
R squared	0.5856	0.7599
Sum of Squares	2.634	1.396
Sy.x	0.2295	0.1845
Number of points		
# of X values	54	54
# Y values analyzed	54	45
**H2009 Cells**		
**log(inhibitor) vs. response -- Variable slope (four parameters)**		
Best-fit values	**MGS4_V8**	**MGS4_V9**
Bottom	0.2419	0.2785
Top	0.8980	0.9256
LogIC50	-7.644	-7.403
HillSlope	-0.8971	-0.7054
IC50	2.271e-008	3.956e-008
Span	0.6561	0.6471
95% CI (asymptotic)		
Bottom	-0.3147 to 0.7984	-0.7242 to 1.281
Top	0.7677 to 1.028	0.7965 to 1.055
LogIC50	-8.829 to -6.459	-9.678 to -5.127
HillSlope	-2.343 to 0.5492	-2.062 to 0.6513
IC50	1.483e-009 to 3.478e-007	2.097e-010 to 7.462e-006
Span	0.05284 to 1.259	-0.4068 to 1.701
Goodness of Fit		
Degrees of Freedom	32	32
R squared	0.5403	0.4908
Sum of Squares	1.553	1.296
Sy.x	0.2203	0.2013
Number of points		
# of X values	36	36
# Y values analyzed	36	36

Supplementary Table 8. Total Radiant Efficiency Measurements for Tumors of Individual Animals: Data Set for Figure 5a						
**MGS4_V8**						
**Time (H)**	**Mouse 1**	**Mouse 2**	**Mouse 3**	**Mouse 4**	**Mean**	**SEM**
12	2.24e+010	1.58e+010	1.1e+010	1.38e+010	1.58e+010	2.43e+009
24	1.78e+010	1.16e+010	1.07e+010	1.31e+010	1.33e+010	1.58e+009
48	1.18e+010	5.34e+009	9.18e+009	5.3e+009	7.91e+009	1.59e+009
72	1.09e+010	3.4e+009	9.02e+009	4.66e+009	7.00e+009	1.77e+009
72	2.44e+010	1.32e+010	2.54e+010	1.78e+010	2.02e+010	2.88e+009
**MGS4_V6**						
**Time (H)**	**Mouse 1**	**Mouse 2**	**Mouse 3**	**Mouse 4**	**Mean**	**SEM**
12	3.81e+008	1.14e+008	7.39e+008	3.43e+008	3.94e+008	1.29e+008
24	3.11e+008	1.18e+008	6.83e+008	2.65e+008	3.44e+008	1.20e+008
48	1.36e+008	1.07e+008	5.03e+008	3.1e+008	2.64e+008	9.14e+007
72	2.58e+008	9.39e+007	4.78e+008	2.88e+008	2.80e+008	7.87e+007
72	1.82e+009	3.14e+009	1.53e+009	1.54e+009	2.00e+009	3.83e+008
**Untreated**						
**Time (H)**	Mouse 1	Mouse 2	Mouse 3	Mouse 4	**Mean**	**SEM**
12	2.09e+008	9.95e+007	1.46e+008	7.05e+007	1.31e+008	3.02e+007
24	6.23e+007	1.35e+008	8.43e+007	1.7e+008	1.13e+008	2.44e+007
48	1.55e+008	7.12e+007	1.28e+008	7.38e+007	1.07e+008	2.08e+007
72	1.31e+008	1.17e+008	1.49e+008	1.11e+008	1.27e+008	8.45e+006
72	2.02e+009	1.88e+009	**	3.44e+008	1.41e+009	5.37e+008
*** Measurement not made due to experimental error in isolating the tumor*						

Supplementary Table 9. Tumor Volumes for Individual Animals: Data Set for Figure 5c										
**Tumor Measurements MGS4_V8-Saporin: Individual Animals**										
**Day**	**0**	**2**	**4**	**6**	**8**	**10**	**12**	**14**	**16**	**18**
Mouse 1	250	256	387	211	229	295	214	211	238	266
Mouse 2	422	429	379	437	430	356	425	476	603	528
Mouse 3	69	88	58	32	12	12	9	4	3	2
Mouse 4	37	47	72	61	74	79	52	74	67	95
Mouse 5	71	113	112	100	93	117	146	109	110	111
Mouse 6	69	97	157	232	300	497	384	572	390	441
Mouse 7	83	156	232	219	244	338	499	331	418	816
Mouse 8	253	221	235	265	299	419	317	461	683	472
Mouse 9	161	173	281	206	161	344	400	248	335	576
**Average**	157	176	213	196	205	273	272	276	316	367
**SEM**	40	36	39	38	41	52	55	62	74	84
**Tumor Measurements MGS4_V6-Saporin: Individual Animals**										
**Day**	**0**	**2**	**4**	**6**	**8**	**10**	**12**	**14**	**16**	**18**
Mouse 1	114	131	138	488	253	341	442	747	1086	466
Mouse 2	78	115	184	246	342	389	630	535	700	717
Mouse 3	199	150	222	266	381	458	493	424	463	660
Mouse 4	109	266	352	268	600	753	946	1153	732	1203
Mouse 5	283	259	504	450	386	454	537	572	578	886
Mouse 6	188	238	280	301	296	267	731	900	370	301
Mouse 7	79	120	154	260	580	243	298	301	334	1029
Mouse 8	69	181	234	206	268	687	464	514	1067	533
**Average**	140	182	259	311	388	449	568	643	666	724
**SEM**	25	21	40	34	44	61	66	91	96	100
**Tumor Measurements Untreated: Individual Animals**										
**Day**	**0**	**2**	**4**	**6**	**8**	**10**	**12**	**14**	**16**	**18**
Mouse 1	157	165	359	426	441	756	911	503	237	586
Mouse 2	252	163	192	202	264	164	144	285	549	596
Mouse 3	114	241	281	404	704	621	618	692	983	1251
Mouse 4	143	193	285	347	409	579	747	950	647	235
Mouse 5	139	310	247	298	275	419	342	244	929	750
Mouse 6	88	134	150	308	479	699	683	454	318	1062
Mouse 7	37	253	248	282	241	310	463	801	579	479
Mouse 8	48	186	199	513	352	556	536	454	507	556
**Average**	122	206	245	348	396	513	556	548	594	689
**SEM**	23	19	22	32	50	67	80	82	87	108

Supplemental Figure 1. Structure of monomeric, dimeric, and tetrameric cores used to display the MGS4 peptide variants.

a) Unless noted in the text, a biotin tag was incorporated into core of the peptides allowing for the use of multiple streptavidin reagents: streptavidin-Alexa Fluor 647, streptavidin-Alexa Fluor 555, streptavidin -Qdot605, streptavidin-R-phycoerythrin, and streptavidin-saporin. b) For NIR imaging experiments, Alexa Fluor 750 was directly conjugated to the peptide by the reaction of maleimide modified dye with a thiol containing MGS4_V8 peptide. c) The structure of the monomeric MGS4 used for serum stabilities. Additional tags or labeled were avoided as to not complicate identification of the potential degradation products.

Supplemental Figure 2. MGS4_V8 is serum stable

MGS4_V4 (non-acetylated, panel a) and MGS4_V8 (acetylated, panel b) were incubated in 100% human serum for 0 and 48-hours. Serum proteins were precipitated, and the peptide was analyzed by analytical HPLC. The mass of the peptide and degradation products were determined by MALDI MS and are indicated in the figure.

Supplemental Figure 3. EC50 of monomeric, dimeric, and tetrameric MGS4 variants on three NSCLC cell lines

MGS4_V8, MGS4_V9, or MGS4_V10 were conjugated with streptavidin-Alexa Fluor 650 and were incubated with the indicated cell line for 1 hour. Non-internalized peptide was removed, and the mean number of peptides internalized per cell was determined to calculate the EC50. Error bars represent standard error measurements and are below the height of the symbols in some cases.

Supplemental Figure 4: Peptide-Streptavidin conjugates are nontoxic to cells.

a. MGS4_V8 and MGS4_V9 were conjugated to streptavidin without saporin. H1299 cells were incubated with peptide-streptavidin complex at indicated concentrations for 6 hours, then removed and complete growth media returned to the wells. After 72 hours, viability was measured. No reduction in cell viability is observed. b. In the same fashion, the inactive MGS4_V6 was conjugated to saporin and incubated with H1299, H2009, and HBEC cells. Fifty percent cell death was not reached at 200 nM. Similarly, MGS4_V8-saporin shows dramatically reduced activity on the control HBEC cell line compared to H1299 and H2009 cells.

Supplemental Figure 5. MGS4_V8 homes to H1299 xenograft tumor after systemic delivery.

H1299 tumor bearing nude mice (N=4) were injected I.V. with MGS4_V8 or MGS4_V6 conjugated to NIR dye Alexa Fluor 750. At 24, 48, and 72 hours post injection, mice were anesthetized and imaged on an IVIS® (Perkin Elmer) to measure total radiant efficiency in each tumor. MGS4_V8 accumulates in tumor ≈3-fold better than control peptide, MGS4_V6. MGS4_V6 accumulation is statistically no different than untreated tumors. Total radiant efficiency measurement for individual animals is shown below the bar graph.

Supplemental Figure 6. Sample Flow Cytometry Data: Establishing a Standard Curve for MESF vs. MFI.

A standard curve was generated using Quantum™ Alexa Fluor 647 microspheres (Bangs Laboratory, Fishers, IN). A representative linear regression showing the correlation of molecule equivalents soluble fluorophores (MESF) vs the mean fluorescence intensity (MFI) is shown. The MFI is determined at 50% peak height.

Supplemental Figure 7. Example of flow cytometry data: MGS4_V10 Uptake on H1993 Cells.

The gating strategy, cell population data , and MFI determination for an example binding experiment is shown. Cells were gated by forward and side scatter to exclude nonviable cells or cell clusters as shown. Individual dot plots and histograms are shown for a concentration range of 0.1 nM – 50 nM. The MFI is determined at 50% peak height. Molecules internalized per cell were determined by the standard curve relating MESF to MFI and divided by the number dye molecules/MGS conjugate.

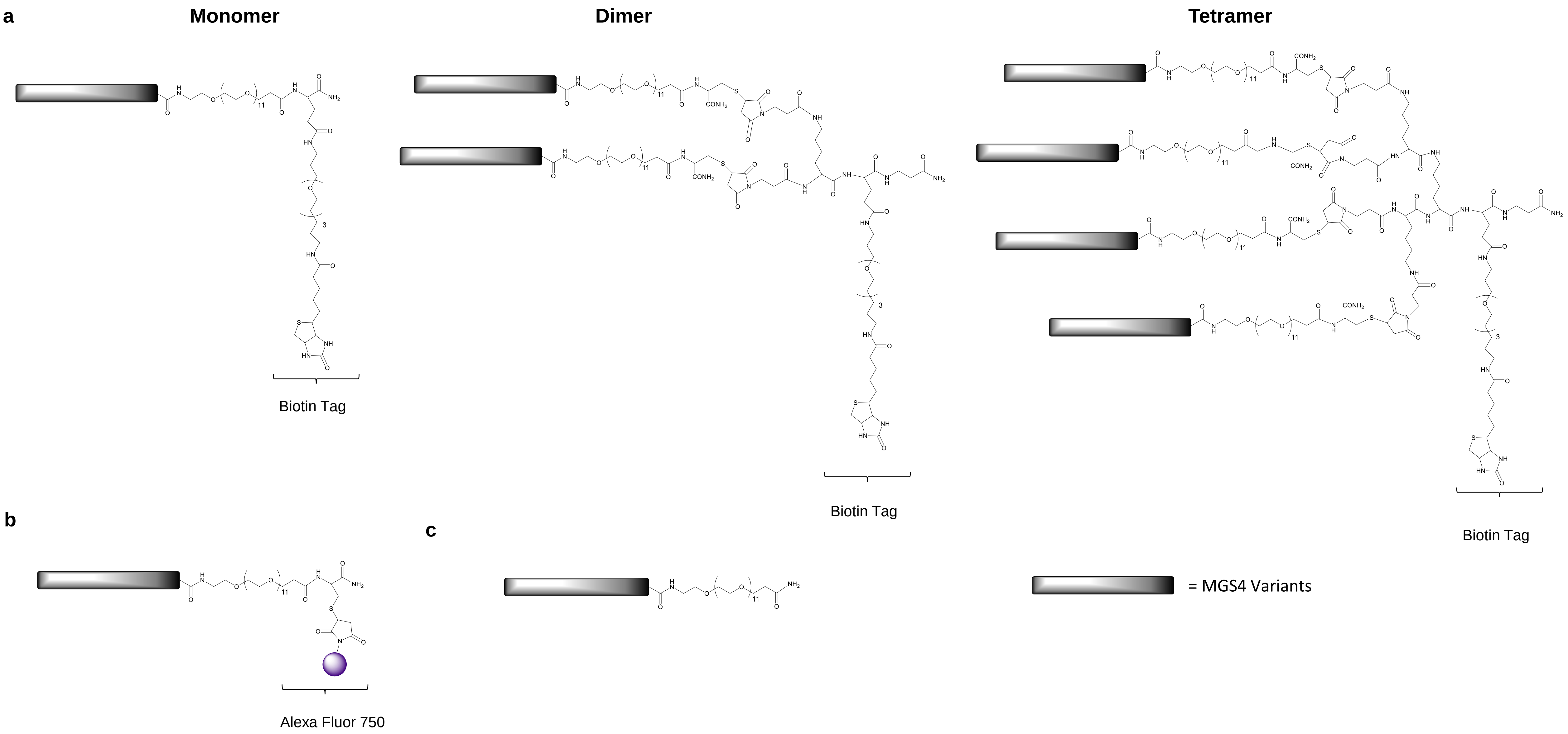
a
Monomer
Dimer
Tetramer
Biotin Tag
Biotin Tag
Biotin Tag
b
Alexa Fluor 750
c
= MGS4 Variants

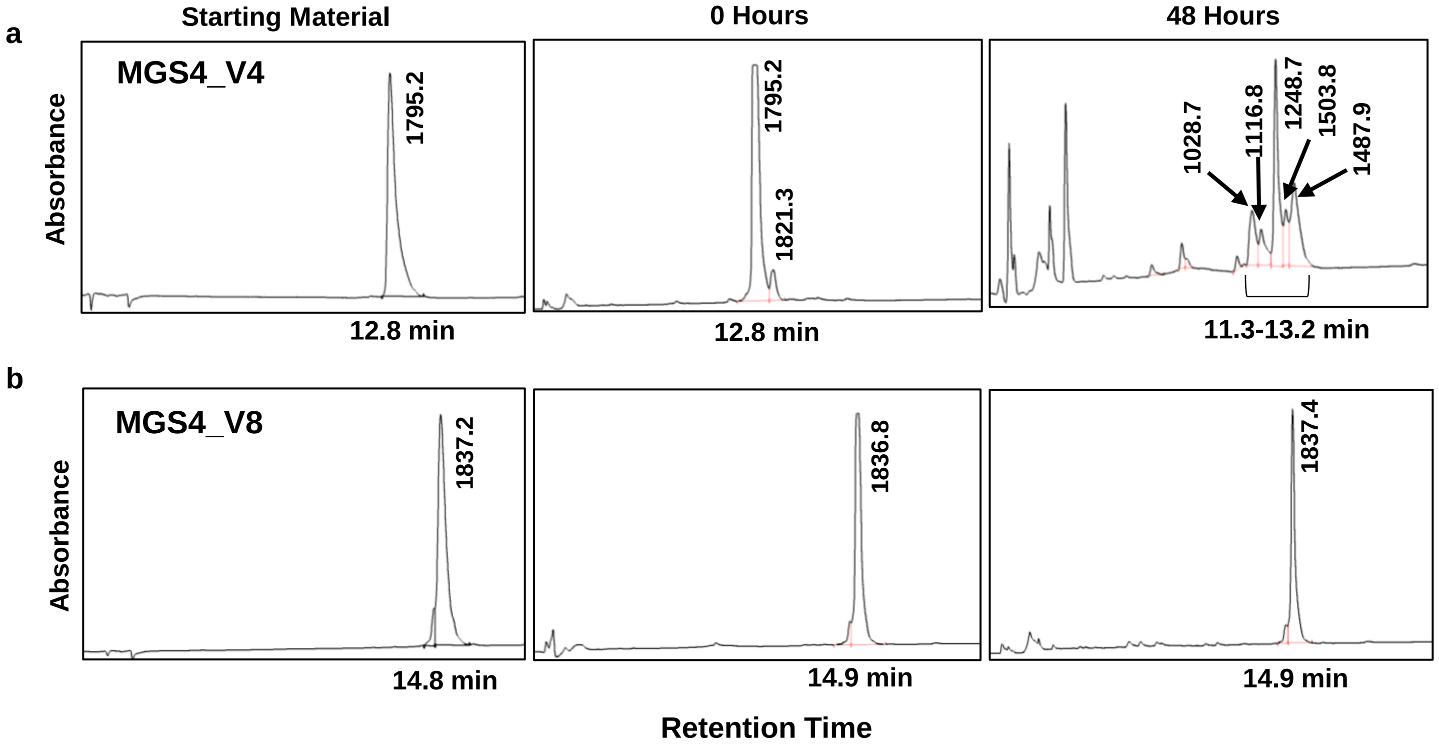

a
b
Starting Material
0 Hours
48 Hours
MGS4_V4
MGS4_V8
Absorbance
Absorbance
1795.2
1795.2
1821.3
1028.7
1116.8
1248.7
1503.8
1487.9
12.8 min
12.8 min
11.3-13.2 min
1837.2
1836.8
1837.4
14.8 min
14.9 min
14.9 min
Retention Time

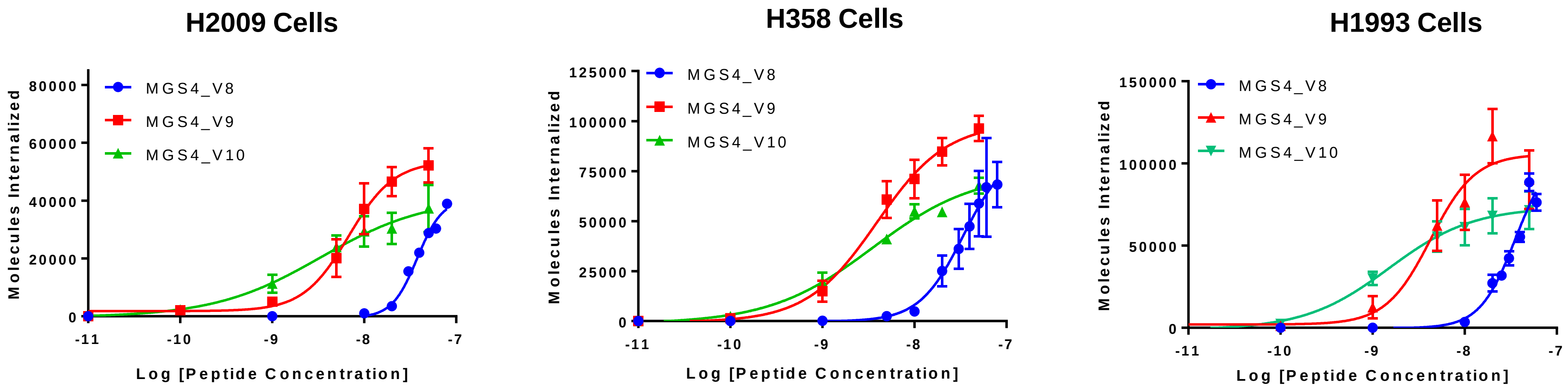
H2009 Cells
H358 Cells
H1993 Cells
MGS4_V8
MGS4_V9
MGS4_V10
Molecules Internalized
Log [Peptide Concentration]
80000
60000
40000
20000
0
125000
100000
75000
50000
25000
150000
-11
-10
-9
-8
-7

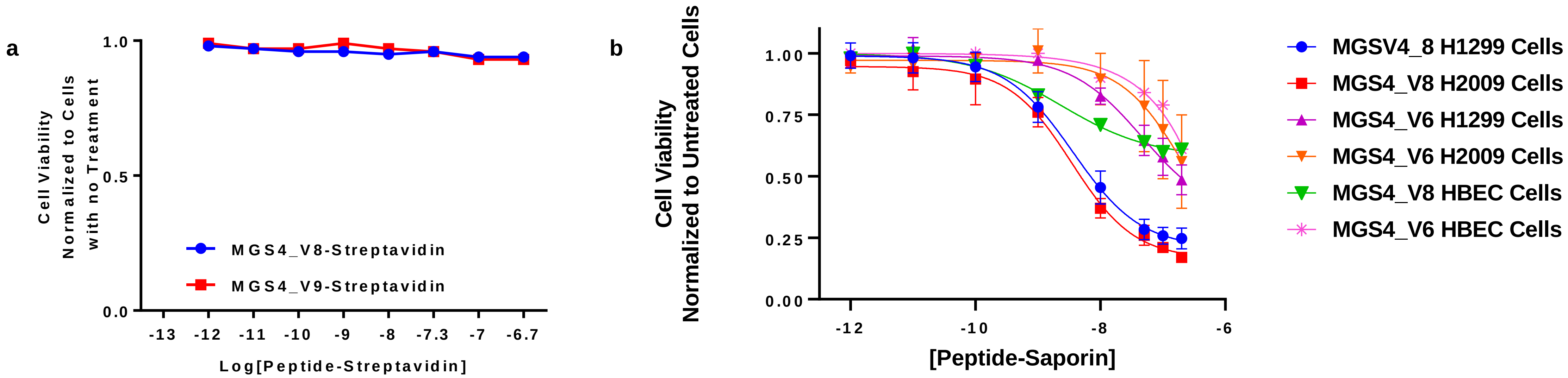
a
Cell Viability
Normalized to Cells
with no Treatment
1.0
0.5
0.0
MGS4_V8-Streptavidin
MGS4_V9-Streptavidin
-13
-12
-11
-10
-9
-8
-7.3
-7
-6.7
Log[Peptide-Streptavidin]
b
Cell Viability
Normalized to Untreated Cells
1.00
0.75
0.50
0.25
0.00
-12
-10
-8
-6
[Peptide-Saporin]
MGSV4_8 H1299 Cells
MGS4_V8 H2009 Cells
MGS4_V6 H1299 Cells
MGS4_V6 H2009 Cells
MGS4_V8 HBEC Cells
MGS4_V6 HBEC Cells

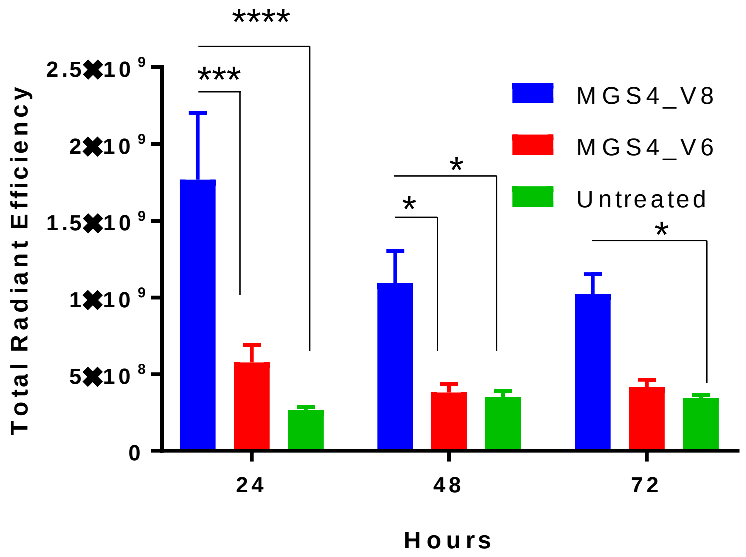

	MGS4_V8				MGS4_V6				Untreated			
Hours	Mouse 1	Mouse 2	Mouse 3	Mouse 4	Mouse 1	Mouse 2	Mouse 3	Mouse 4	Mouse 1	Mouse 2	Mouse 3	Mouse 4
24	1.32e+009	1.83e+009	9.57e+008	2.96e+009	7.74e+008	4.6e+008	7.58e+008	3.13e+008	3.17e+008	2.76e+008	2.2e+008	2.56e+008
48	9.13e+008	9.76e+008	7.72e+008	1.71e+009	5.44e+008	3.19e+008	3.39e+008	3.19e+008	4.21e+008	2.37e+008	3.49e+008	3.94e+008
72	9.42e+008	7.37e+008	1.06e+009	1.35e+009	5.46e+008	3.28e+008	3.61e+008	4.28e+008	3.81e+008	3.06e+008	3.15e+008	3.75e+008

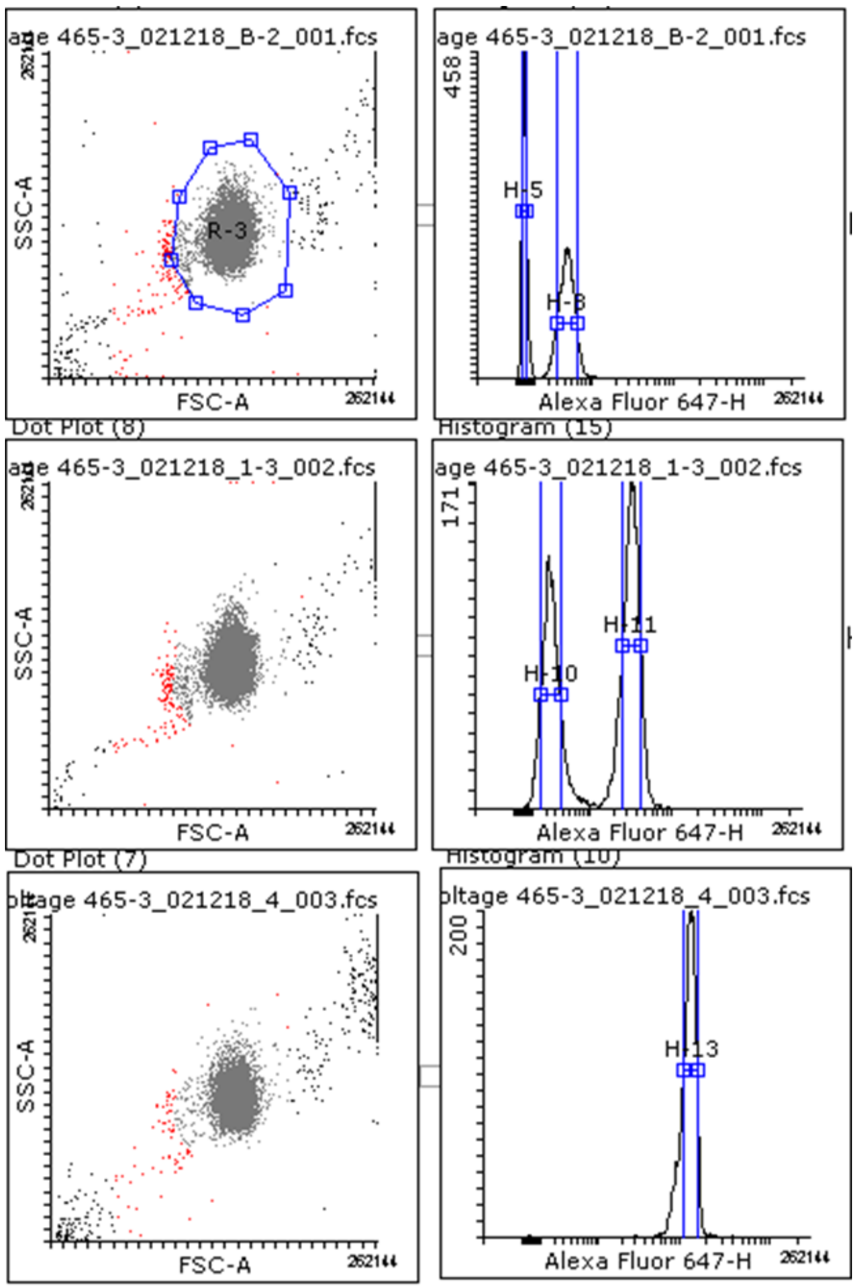

Blank (H-5), Bead # 2 (H-8)

	Events	% of Vis	GeoMean
All events	9044	100	157
H-5	2380	26.3	38
H-8	4351	48.1	429

Bead # 1 (H-10), Bead # 3(H-11)

	Events	% of Vis	GeoMean
All events	9381	100	1072
H-10	3015	32.1	239
H-11	3842	41.0	3391

Bead # 3(H-13)

	Events	% of Vis	GeoMean
All events	4899	100	12570
H-13	3515	71.7	13479

Grey= gated population (Gate shown in the blue circle)
MFI determined at 50% peak height as represented on the histograms

Standardization beads

Beads	MESF	MFI
Blk	0	38
1	13868	239
2	34785	429
3	275843	3391
4	962854	13479

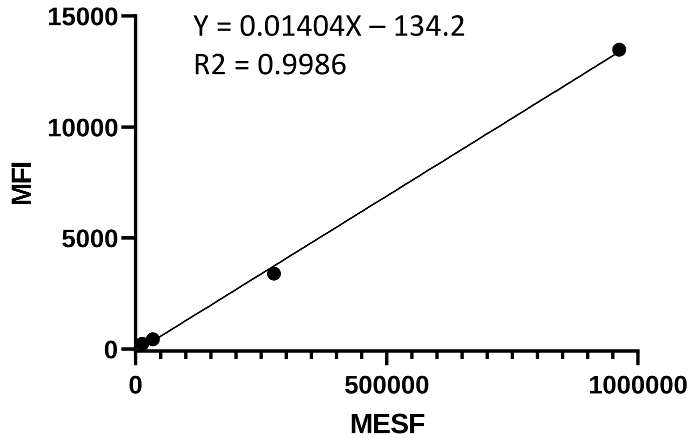

MESF = molecule equivalents soluble fluorophore
MFI = mean fluorescent intensity

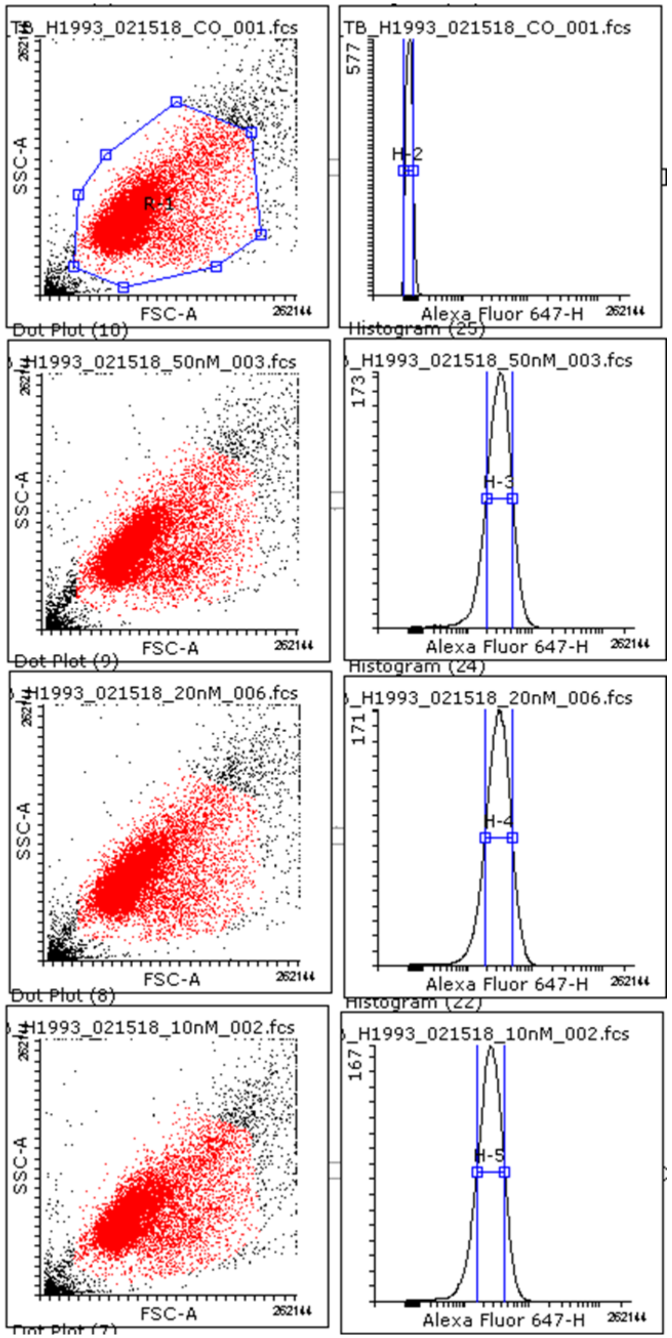

Cells Only	Events	% of Vis	GeoMean
All events	9945	100	33
H-2	9627	97	32

50 nM	Events	% of Vis	GeoMean
All events	10022	100	3067
H-3	7277	73	3281

20 nM	Events	% of Vis	GeoMean
All events	10024	100	2968
H-4	7512	75	3191

10 nM	Events	% of Vis	GeoMean
All events	9983	100	2497
H-5	7368	74	2688

Red= gated population (Gate shown in the blue circle)
MFI determined at 50% peak height as represented on the histograms

Note: Molecules internalized per cell were determined by the standard curve relating MESF to MFI and divided by the number dye molecules/MGS conjugate

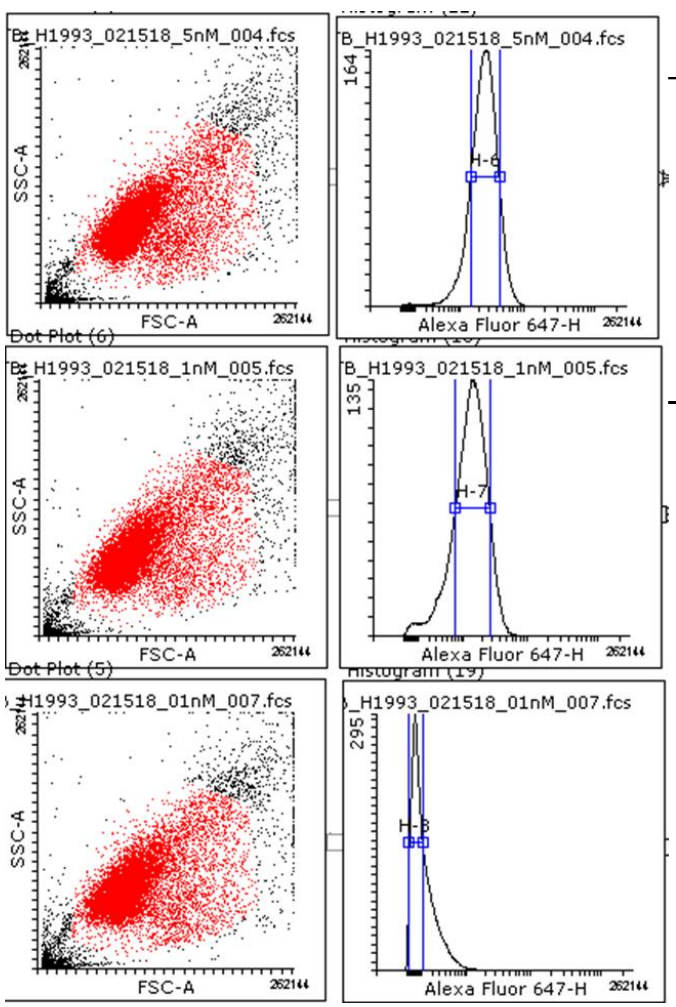

5 nM	Events	% of Vis	GeoMean
All events	9961	100	2378
H-6	7515	75	2588

1 nM	Events	% of Vis	GeoMean
All events	9989	100	1204
H-7	7401	75	1435

0.1nM	Events	% of Vis	GeoMean
All events	10004	100	85
H-8	6310	63	49

Concentration (nM) MGS4_V10	Average Molecules/Cell
0.1	2359
1	27099
5	47693
10	49481
20	58461
50	60071
