# Supplementary material for: Tumor-specific intracellular delivery: peptide-guided transport of a catalytic toxin
Source: Commun Biol. 2023 Jan 17;6:60. doi: 10.1038/s42003-022-04385-7 (PMC9845330; doi:10.1038/s42003-022-04385-7)
Supplement: Supplementary file 2 — Description of Additional Supplementary Files [file 42003_2022_4385_MOESM2_ESM.pdf]

Description of Additional Supplementary Files

File name: Supplementary Data 1
Description: Contains source data for the graphs in the paper (binding assays and in vitro cytotoxicity assays).
