# Supplementary material for: Tumor-specific intracellular delivery: peptide-guided transport of a catalytic toxin
Source: Commun Biol. 2023 Jan 17;6:60. doi: 10.1038/s42003-022-04385-7 (PMC9845330; doi:10.1038/s42003-022-04385-7)
Supplement: Supplementary file 4 — Reporting Summary [file 42003_2022_4385_MOESM4_ESM.pdf]

nature research

Corresponding author(s): Kathlynn C Brown

Last updated by author(s): Dec 9, 2022

Reporting Summary

Nature Research wishes to improve the reproducibility of the work that we publish. This form provides structure for consistency and transparency in reporting. For further information on Nature Research policies, see our Editorial Policies and the Editorial Policy Checklist.

Statistics

For all statistical analyses, confirm that the following items are present in the figure legend, table legend, main text, or Methods section.

n/a	Confirmed	
☐	☒	The exact sample size (*n*) for each experimental group/condition, given as a discrete number and unit of measurement
☐	☒	A statement on whether measurements were taken from distinct samples or whether the same sample was measured repeatedly
☐	☒	The statistical test(s) used AND whether they are one- or two-sided<br>*Only common tests should be described solely by name; describe more complex techniques in the Methods section.*
☒	☐	A description of all covariates tested
☒	☐	A description of any assumptions or corrections, such as tests of normality and adjustment for multiple comparisons
☐	☒	A full description of the statistical parameters including central tendency (e.g. means) or other basic estimates (e.g. regression coefficient) AND variation (e.g. standard deviation) or associated estimates of uncertainty (e.g. confidence intervals)
☒	☐	For null hypothesis testing, the test statistic (e.g. *F*, *t*, *r*) with confidence intervals, effect sizes, degrees of freedom and *P* value noted<br>*Give P values as exact values whenever suitable.*
☒	☐	For Bayesian analysis, information on the choice of priors and Markov chain Monte Carlo settings
☒	☐	For hierarchical and complex designs, identification of the appropriate level for tests and full reporting of outcomes
☒	☐	Estimates of effect sizes (e.g. Cohen's *d*, Pearson's *r*), indicating how they were calculated

Our web collection on statistics for biologists contains articles on many of the points above.

Software and code

Policy information about availability of computer code

Data collection	*Provide a description of all commercial, open source and custom code used to collect the data in this study, specifying the version used OR state that no software was used.*
Data analysis	GraphPad PRISM, Flowing Software

For manuscripts utilizing custom algorithms or software that are central to the research but not yet described in published literature, software must be made available to editors and reviewers. We strongly encourage code deposition in a community repository (e.g. GitHub). See the Nature Research guidelines for submitting code & software for further information.

Data

Policy information about availability of data

All manuscripts must include a data availability statement. This statement should provide the following information, where applicable:

- Accession codes, unique identifiers, or web links for publicly available datasets
- A list of figures that have associated raw data
- A description of any restrictions on data availability

All data generated or analyzed during this study are included in this published article (and its supplementary information files).

Field-specific reporting

Please select the one below that is the best fit for your research. If you are not sure, read the appropriate sections before making your selection.

☒ Life sciences ☐ Behavioural & social sciences ☐ Ecological, evolutionary & environmental sciences

For a reference copy of the document with all sections, see nature.com/documents/nr-reporting-summary-flat.pdf

Life sciences study design

All studies must disclose on these points even when the disclosure is negative.

Sample size	For in vivo imaging, we used 4 animals per group based on our previous experience with small animal imaging.
Data exclusions	No data were excluded.
Replication	All attempts at replication were successful.
Randomization	Animals were grouped so that the average starting size of the tumor was similiar between the groups.
Blinding	Tumors were measured by an independent researcher with no knowledge of the treatment groups.

Reporting for specific materials, systems and methods

We require information from authors about some types of materials, experimental systems and methods used in many studies. Here, indicate whether each material, system or method listed is relevant to your study. If you are not sure if a list item applies to your research, read the appropriate section before selecting a response.

Materials & experimental systems

n/a	Involved in the study	
☐	☒	Antibodies
☐	☐	Eukaryotic cell lines
☒	☐	Palaeontology and archaeology
☐	☒	Animals and other organisms
☒	☐	Human research participants
☒	☐	Clinical data
☒	☐	Dual use research of concern

Methods

n/a	Involved in the study	
☒	☐	ChIP-seq
☐	☒	Flow cytometry
☒	☐	MRI-based neuroimaging

Antibodies

Antibodies used	anti-saporin rabbit polyclonal antibody AB-41AP (Advanced Targeting Systems Bio, San Diego, CA)
Validation	Antibody was validated by the company. No further validation was performed.

Eukaryotic cell lines

Policy information about cell lines

Cell line source(s)	H2009, H1299, H358 and H1993 cells were obtained from the original depositors, John Minna and Adi Gazdar (UT Southwestern Medical Center)
Authentication	Cells were authenticated by genotyping performed by Bio-synthesis (Lewisville, TX)
Mycoplasma contamination	Cells were tested monthly for mycoplasma using MycoAlert Detection Kit manufactured by Lonza
Commonly misidentified lines (See ICLAC register)	None

Animals and other organisms

Policy information about studies involving animals; ARRIVE guidelines recommended for reporting animal research

Laboratory animals	Female Nu/Nu mice 6-10 weeks of age purchased from Jackson Laboratory

Wild animals	None
Field-collected samples	None
Ethics oversight	Animal experiments were approved by SRI International's Institutional Animal Care and Use committee (Animal Welfare Assurance Number A3025-01, protocol 14008).

Note that full information on the approval of the study protocol must also be provided in the manuscript.

Flow Cytometry

Plots

Confirm that:

☒ The axis labels state the marker and fluorochrome used (e.g. CD4-FITC).

☒ The axis scales are clearly visible. Include numbers along axes only for bottom left plot of group (a 'group' is an analysis of identical markers).

☒ All plots are contour plots with outliers or pseudocolor plots.

☒ A numerical value for number of cells or percentage (with statistics) is provided.

Methodology

Sample preparation	Tumor cells were grown to 90% confluency in a 12 well plate, then incubated with 500 µl peptide-dye conjugate at 37oC. After 1 hour, peptide was removed, and the cells were washed 3x with PBS (137 mM NaCl, 2.7 mM KCl, 10 mM Na2HPO4, 1.8 mM KH2PO4, pH 7.4), 2x with 0.1 M HCl-glycine pH2.2 in 0.9% NaCl, and 1x PBS rinse. Cells were removed by trypsinization.
Instrument	BD FACSCelesta
Software	Flowing Software
Cell population abundance	*Describe the abundance of the relevant cell populations within post-sort fractions, providing details on the purity of the samples and how it was determined.*
Gating strategy	Cells were gated based on the forward and side scatter to include only viable cells and a minimum of 10,000 events were counted. A region containing < 5% of the cells in the negative control was established.

☒ Tick this box to confirm that a figure exemplifying the gating strategy is provided in the Supplementary Information.
